# Unravelling the molecular mechanism underlying drought stress response in chickpea *via* integrated multi-omics analysis

**DOI:** 10.3389/fpls.2023.1156606

**Published:** 2023-05-23

**Authors:** Vikram Singh, Khushboo Gupta, Shubhangi Singh, Mukesh Jain, Rohini Garg

**Affiliations:** ^1^ School of Computational & Integrative Sciences, Jawaharlal Nehru University, New Delhi, India; ^2^ Department of Life Sciences, Shiv Nadar Institution of Eminence, Gautam Buddha Nagar, Uttar Pradesh, India

**Keywords:** chickpea, drought, metabolome, proteome, transcriptome, co-expression, WGCNA

## Abstract

Drought stress affects growth and productivity significantly in chickpea. An integrated multi-omics analysis can provide a better molecular-level understanding of drought stress tolerance. In the present study, comparative transcriptome, proteome and metabolome analyses of two chickpea genotypes with contrasting responses to drought stress, ICC 4958 (drought-tolerant, DT) and ICC 1882 (drought-sensitive, DS), was performed to gain insights into the molecular mechanisms underlying drought stress response/tolerance. Pathway enrichment analysis of differentially abundant transcripts and proteins suggested the involvement of glycolysis/gluconeogenesis, galactose metabolism, and starch and sucrose metabolism in the DT genotype. An integrated multi-omics analysis of transcriptome, proteome and metabolome data revealed co-expressed genes, proteins and metabolites involved in phosphatidylinositol signaling, glutathione metabolism and glycolysis/gluconeogenesis pathways, specifically in the DT genotype under drought. These stress-responsive pathways were coordinately regulated by the differentially abundant transcripts, proteins and metabolites to circumvent the drought stress response/tolerance in the DT genotype. The *QTL-hotspot* associated genes, proteins and transcription factors may further contribute to improved drought tolerance in the DT genotype. Altogether, the multi-omics approach provided an in-depth understanding of stress-responsive pathways and candidate genes involved in drought tolerance in chickpea.

## 1 Introduction

Chickpea (*Cicer arietinum* L.) is the second most important grain legume cultivated worldwide, especially in developing countries. India ranks first in chickpea production accounting for approximately 75% of the total production (FAO, 2016). Chickpea has high nutritional value containing proteins (20–25%), carbohydrates (61.5%) and fatty acids (4.5%), along with a significant amount of essential amino acids and vitamin content (Jukanti et al., 2012). Chickpea is cultivated mainly in arid and semi-arid regions with limited water availability. Therefore, drought stress is a major constraint that causes significant loss in growth and productivity of chickpea. Chickpea employs an adaptive strategy to tolerate limited drought stress due to its well-established root system architecture. Significant phenotypic variations in root characteristics have been reported among various chickpea genotypes. Higher biomass, root length density and better root system architecture are the characteristics of drought-tolerant (DT) genotypes, whereas drought-sensitive (DS) genotypes exhibit reduced root characteristics (Gaur et al., 2008; Kashiwagi et al., 2015).

The implementation of different high-throughput “omics” techniques in silos, including transcriptomics, proteomics and metabolomics have enabled the understanding of drought stress responses in chickpea to some extent (Garg et al., 2016; Khan et al., 2019; Bhaskarla et al., 2020; Gupta et al., 2020). A few studies have been performed to elucidate the global transcriptional dynamics to identify the genes and regulatory networks under various abiotic stress conditions (Garg et al., 2015; Garg et al., 2016; Kudapa et al., 2018; Mashaki et al., 2018; Kumar et al., 2019). The transcriptome analysis of chickpea roots under drought stress for different DT and DS genotypes revealed the involvement of various transcription factors (TFs), metabolic pathways and biological processes in the drought stress responses (Garg et al., 2016; Bhaskarla et al., 2020). The proteomics approaches have also been employed to examine the complexity of drought stress response. The proteins localized in different cellular compartments and those involved in different metabolic processes were found to contribute to drought tolerance in chickpea (Barua et al., 2019; Cevik et al., 2019; Gupta et al., 2020; Vessal et al., 2020). However, limited progress has been made towards metabolome analysis in chickpea under stress conditions. The metabolite profiling of leaves of two chickpea genotypes, Noor‐2009 (drought-sensitive) and 93127 (drought-tolerant) revealed the changes in amino acids composition under drought stress (Khan et al., 2019).However, these studies employed a stand-alone omics approach to investigate the drought stress response in chickpea. The integration of transcriptome, proteome and/or metabolome data have provided an impressive understanding of biotic, abiotic and oxidative stress response, nitrogen metabolism, tiller production, alkaloids biosynthesis, iron homeostasis and fruit ripening in different plants (Amiour et al., 2012; Srivastava et al., 2013; Sudre et al., 2013; Zeng et al., 2013; Remmers et al., 2018; Li et al., 2019a; Wang et al., 2019; Yun et al., 2019; Chin et al., 2020; Moreno et al., 2021; Bittencourt et al., 2022; Leão et al., 2022; Shu et al., 2022). An integrated analysis of transcriptome, proteome and metabolome in different chickpea genotypes can provide a better understanding of molecular mechanisms and pathways underlying drought stress response/tolerance.

In this study, we used two chickpea genotypes with contrasting responses to drought stress, namely ICC 4958 (drought-tolerant, DT) and ICC 1882 (drought-sensitive, DS). These chickpea genotypes have been characterized extensively for their contrasting responses to drought stress and used for the generation of bi-parental populations for the QTL mapping (Jaganathan et al., 2015). These genotypes showed different root traits, phenotypic responses and yield under drought stress (Kashiwagi et al., 2005; Krishnamurthy et al., 2010; Kashiwagi et al., 2013; Garg et al., 2016; Purushothaman et al., 2017). ICC 4958 showed higher root and shoot dry weight, and larger roots in comparison to ICC 1882 (Garg et al., 2016). Moreover, higher shoot biomass, grain yield and harvest index were also observed for ICC 4958 under drought stress (Purushothaman et al., 2016). Here, we analyzed the transcriptome, proteome and metabolome of the roots of ICC 4958 and ICC 1882 genotypes under control and drought stress conditions to gain mechanistic insights into the drought stress tolerance in chickpea. The differential expression analyses identified the differentially abundant transcripts, proteins and metabolites under control and/or drought stress conditions in/between the chickpea genotypes. Weighted gene co-expression network analysis (WGCNA) was performed to explore the co-expressed genes, proteins and metabolites. The co-expressed genes/modules were further investigated for the identification of TF encoding genes, and those located within the known *QTL*-*hotspot* region for drought tolerance in chickpea. The integrated analysis of transcriptome, proteome and metabolome data revealed a crucial role of phosphatidylinositol (PI) signaling, glutathione metabolism and glycolysis/gluconeogenesis pathways in drought tolerance. These results provide new insights into the drought tolerance mechanism in chickpea and prioritization of candidate genes for functional analysis.

## 2 Materials and methods

### 2.1 Plant materials and drought stress treatment

The seeds of two chickpea genotypes with contrasting response to drought stress, ICC 4958 (drought-tolerant, DT) and ICC 1882 (drought-sensitive, DS) were procured from International Crops Research Institute for the Semi-arid Crops (ICRISAT), Hyderabad, India. The seeds of both genotypes were grown in pots containing soilrite saturated with reverse osmosis water. The pots were kept in a plant growth chamber with day (28°C in 400 µmol photons/m^2^/s of light intensity) and night (23°C in dark) cycle of 14 h and 10 h, respectively. Drought stress was imposed on 11 d old plants by withholding water for 15 d, while control seedlings were watered on alternate days until the end of the experiment. Water withholding for different days has been used for imposing drought stress in previous studies (Garg et al., 2016; Sinha et al., 2019). We observed significant difference in the root growth of DT and DS genotypes under drought stress after 15 d and collected the root samples from the control and drought-stressed plants of both genotypes to investigate the molecular changes. The samples were harvested in multiple technical replicates, snap frozen in liquid nitrogen and stored at -80 °C until further analysis. The root tissues were harvested in three independent biological replicates. The root tissues harvested from the same experiments were used for transcriptome, proteome and metabolome analyses.

### 2.2 RNA sequencing and data pre-processing

Total RNA from each sample was extracted using TRI reagent according to the manufacturer’s instructions (Sigma Life Sciences). RNA quality and quantity were assessed using Nanodrop Spectrophotometer and Agilent Bioanalyzer (Agilent technologies, Singapore) as described previously (Garg et al., 2010). Further, libraries from two biological replicates of each sample were prepared and sequencing was performed on the Illumina platform to generate 49-nt single-end reads. Quality control of raw reads was performed using NGS QC Toolkit (v2.3.3) (Patel and Jain, 2012) for removal of reads harboring adapter sequence and/or bases with poor quality. The filtered high-quality reads were used for downstream analysis.

#### 2.2.1 Read mapping and differential gene expression analysis

The filtered high-quality reads were mapped on the kabuli chickpea genome (v1.0; Varshney et al., 2013b) using Tophat (v2.1.1) (Trapnell et al., 2009) software. To analyze gene expression, a reference-guided assembly of the transcriptome data from all samples was generated using Cufflinks (v2.2.1) (Trapnell et al., 2012). Cuffmerge was used to create consensus assembly from the reference-guided assemblies generated for each sample. Differential expression analysis was performed for DT and DS genotypes under drought (D) relative to control (C), [DT(D/C)] and [DS(D/C)], respectively, and for DT genotype relative to DS genotype under control (C) conditions, [DT(C)/DS(C)]. The differential expression analysis between different samples was performed using Cuffdiff. The transcripts with *P*-value ≤ 0.05 and log_2_ fold change of ≥ 1 (up-regulated) and ≤ -1 (down-regulated) were considered to be differentially abundant.

### 2.3 Total protein extraction

Grounded root tissue (~1 g) for each sample (in three biological replicates) was suspended into 3 ml of extraction buffer (pH, 8.0) [Tris-HCl (500 mM), EDTA (50 mM), sucrose (700 mM), KCl (100 mM), β-mercaptoethanol (2%) and phenylmethylsulfonyl fluoride (1 mM)], vortexed briefly and incubated in ice for 10 min. An equal volume of Tris-HCl saturated phenol (pH, 6.6–7.9) was added to the sample and kept at room temperature for 10 min. Samples were centrifuged at 12,000 g for 10 min at 4°C and upper phenolic phase was recovered carefully into a new tube. The phenolic phase was once again extracted with 3 ml of extraction buffer as described above. Following centrifugation, the upper phase was carefully recovered and transferred into a new tube. Afterwards, 4 volumes of precipitation solution (0.1 M ammonium acetate in cold methanol) was added. The properly mixed solution was incubated overnight at -20°C. Finally, total proteins were pelleted down by centrifugation at 5500 g for 10 min at 4°C. The protein pellet was dissolved in solubilization buffer [Tris-HCl (1.5 M, pH 8.0) and urea (8 M)] and the solubilized protein sample was used for nanoLC-MS/MS analysis.

#### 2.3.1 Nano-LC-MS/MS and data processing

Each protein sample (25 µl) was reduced using 5 mM tris (2-carboxyethyl)phosphine (TCEP) followed by alkylation with iodoacetamide (50 mM) and digestion with trypsin (1:50 of trypsin/lysate ratio) for 16 h at 37°C. Digested samples were cleaned using C18 silica cartridge to remove the salts and dried using speed vacuum. Dried protein pellet was resuspended in solubilization buffer A [acetonitrile (5%) and formic acid (0.1%)]. NanoLC-MS/MS analysis of the solubilized protein sample was performed using EASY-nLC 1000 system (Thermo Fisher Scientific) coupled to Thermo Q-Exactive equipped with nanoelectrospray ion source by the commercial service provider (Valerian Chem Pvt. Ltd., New Delhi). About 1.0 µg of the digested protein (peptides) was resolved using 60 cm Viper column (360 µm outer diameter, 75 µm inner diameter, 10 µm tip) filled with 3.0 µm of C18-resin. Further, peptides were loaded with buffer A (5% acetonitrile and 0.1% formic acid) and eluted with 0 – 40% gradient of buffer B [acetonitrile (95%) and formic acid (0.1%)] at a flow rate of 300 nl/min for 100 min.

The raw data was analyzed for qualitative and quantitative proteome using MaxQuant (MQ) (version 1.6.14.0) (Tyanova et al., 2016). The collected spectra of all peptides were searched against all the annotated protein sequences (28,269) in the reference chickpea genome (Varshney et al., 2013a) to identify its corresponding proteins using Andromeda, a peptide search engine (Cox et al., 2011). The protease used to generate peptides was set as enzyme specificity for trypsin/P. The estimated false discovery rate (FDR) of all peptide and protein identifications was set to 1%. The mass tolerance was kept to 7 ppm for precursor and fragment ions. Two missed cleavage values were allowed, and the minimum peptide length was set to 7 amino acids. The label-free method was used for protein quantification with classic type normalization and minimum ratio count of two. MQ provided the MS/MS spectrum details of all the identified peptides. Both unique and razor peptides were used for the identification of proteins, and protein quantification (abundance) was referred to their intensities. Differential expression analysis of proteins was performed using edgeR for DT(D/C), DS(D/C) and DT(C)/DS(C) comparisons. Proteins with log_2_ fold change of ≥ 1 (up-regulated) and ≤ -1 (down-regulated) and *P*-value ≤ 0.05 were considered as differentially abundant.

### 2.4 Sample preparation and gas chromatography-mass spectrometry for metabolome analysis

Frozen root samples (three biological replicates of each sample) were grounded into fine powder using liquid nitrogen. About 200 mg of each grounded sample was homogenized with 5 ml of pre-chilled methanol:acetonitrile (2:1) solvent in pestle and mortar. The solution was centrifuged at 9,500 rpm for 15 min at 25°C and supernatant was divided into two aliquots representing the technical replicates. Thereafter, 50 μl of ribitol (2 mg/ml in ddw) was added to each sample to serve as internal standard. The supernatant was dried in a speed vacuum concentrator at 25°C until the sample dryness reached to ~95%. Further, derivatization of samples was done by adding 50 μl of methoxyamine hydrochloride solution (30 mg methoxyamine hydrochloride in 1 ml of pyridine) followed by incubation at 30°C for 90 min. Subsequently, 100 μl of N-methyl-N-trimethyl silyl tri-flouro acetamide (MSTFA) was added to each sample and incubated at 37°C for 60 min for tri-methyl-sialylation (TMS).

Two technical replicates of each derivatized sample (biological replicate) were analyzed by GC-MS (QP2010 ULTRA, Shimadzu, Japan). The derivatized sample (1 μl) was injected into a Rtx-5Sil-MS (30 m × 0.25 mm × 0.25 μm) column using a split injection mode with the sampling time of 1 min, injection temperature 260°C and column oven temperature of 70°C using the flow control mode as linear velocity. Helium was used as a carrier gas with a flow rate of 1 ml min^-1^. The initial oven temperature was set as 70°C for 3 min and then increased to 280°C until full run. The following parameters was adjusted for MS analysis, ion source temperature at 230°C, interference temperature at 270°C, run time of 50 min, scan speed of 3333 and mass by charge ratio (m/z) of 40 – 650. Acquisition of total ion chromatogram (TIC) was done for metabolite identification and quantification *via* GCMS solution 4.20 software (Shimadzu, Japan). Peak integration was performed to calculate the peak area, height and retention time (RT), and identification of metabolites was done by the similarity search of obtained mass spectra with the mass spectra available in NIST14, NIST14s and Wiley08 libraries. The manual curation of all the identified metabolites was performed to retain a unique set of metabolites for each sample analyzed. The metabolites identified in at least three replicates of each sample were used for further analysis and metabolite levels were normalized using ribitol as an internal standard. Differential expression analysis of metabolites was done by edgeR for DT(D/C), DS(D/C) and DT(C)/DS(C) comparisons, and metabolites with log_2_ fold change of ≥1 (up-regulated) and ≤-1 (down-regulated) and *P*-value ≤0.05 were considered as differentially abundant.

### 2.5 Co-expression network analysis

We used WGCNA (v 1.70-3; Langfelder and Horvath, 2008) to perform co-expression analysis for transcriptome, proteome and metabolome data sets for all the four samples, DT(C), DT(D), DS(C) and DS(D). Co-expression analysis was performed by using log_2_ normalized values of FPKM (for 9847 genes showing ≥ 0.1 variance), intensity (for 2430 proteins) and relative amount (for 133 metabolites) of transcriptome, proteome and metabolome data, respectively. The matrix of co-expression data was generated using an optimized beta power (β) of three for transcriptome, seven for proteome and four for metabolome, and transformed into topological overlap matrix (TOM). The hierarchical clustering was performed using an average method to generate dendrogram. Module identification was done by dynamicTreeCut function to generate clusters with minimum size of 30 for transcriptome and proteome, and 15 for metabolome, and deepSplit value of 2. Highly correlated modules were merged at cutHeight of 0.2 to obtain the final set of modules.

### 2.6 Integrated analysis of transcriptome, proteome and metabolome

The integrated analysis of transcriptome, proteome and metabolome data was done using WGCNA as described earlier (Kelly et al., 2018; Liu et al., 2020). Integration of transcriptome, proteome and metabolome data was done by using their module eigenvectors. Two-way integration approach was adopted for the data integration, where transcriptome and proteome data were integrated using eigenvector for co-expressed gene modules (eigengenes) and co-expressed protein modules (eigenproteins) to identify the correlation between eigengenes and eigenproteins. Similarly, integration of transcriptome and metabolome data using eigenvectors for co-expressed gene modules (eigengenes) and co-expressed metabolite modules (eigenmetabolites) was performed to reveal correlation between eigengenes and eigenmetabolites.

### 2.7 GO and pathway enrichment analysis

GO enrichment analysis for differentially abundant transcripts (DATs) and differentially abundant proteins (DAPs) were performed using BiNGO tool in Cytoscape (v3.7). The GO terms with *P*-value ≤ 0.05 were considered as significantly enriched. The pathway enrichment analysis for DATs and DAPs was performed using KOBAS 3.0 (http://kobas.cbi.pku.edu.cn/) considering KEGG, BioCys and PANTHER databases at the *P*-value cut-off of ≤ 0.05. Pathway enrichment analysis for differentially abundant metabolites (DAMs) was performed using MetaboAnalyst (v.3.0) at *P*-value cut-off of ≤ 0.05.

### 2.8 Reverse transcriptase- quantitative polymerase chain reaction analysis

The RT-qPCR analysis was performed to validate the expression levels of selected candidate genes. A total of 17 genes exhibiting differential expression in DS(D/C), DT(D/C) and/or DT(C)/DS(C) comparisons were selected for the analysis. The primers were designed using Primer Express (3.0) software (Thermo Fischer; USA) and are listed in **Table S11**. The differential abundance levels of the selected genes were determined and compared with the RNA-seq data.

## 3 Results

Drought response/tolerance in different chickpea genotypes has been attributed mainly due to the root system architecture and related traits (Gaur et al., 2008; Garg et al., 2016; Purushothaman et al., 2016). The chickpea genotypes used in this study, ICC 4958 (drought-tolerant, DT) and ICC 1882 (drought-sensitive, DS), showed different root traits and have been characterized extensively for their contrasting responses to drought stress (Kashiwagi et al., 2005; Krishnamurthy et al., 2010; Kashiwagi et al., 2013; Jaganathan et al., 2015; Garg et al., 2016; Purushothaman et al., 2017). To gain deeper mechanistic insights into the drought stress tolerance in chickpea, we implemented a multi-omics (transcriptomics, proteomics and metabolomics) approach to analyze the roots of ICC 4958 and ICC 1882 genotypes under control and drought stress conditions.

### 3.1 Differential gene expression profiling and pathway/GO enrichment analysis

**Figure 1:**
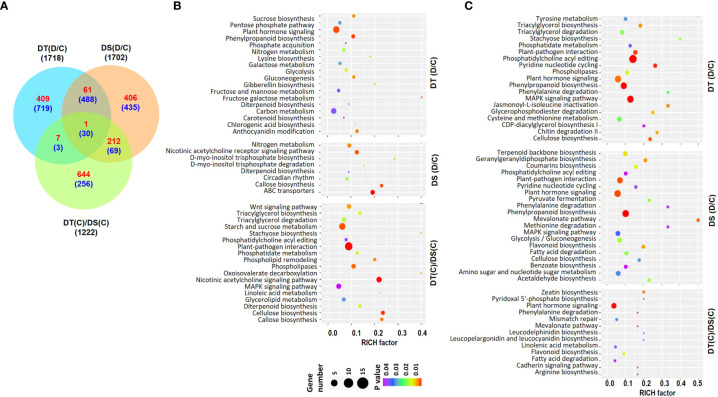
Differentially abundant transcripts (DATs) in drought-tolerant (DT) and drought-sensitive (DS) chickpea genotypes under control (C) and drought stress (D) conditions. DATs in the DT and DS genotypes were identified under drought stress as compared to control, DT(D/C) and DS(D/C), respectively, and between DT and DS genotypes under control condition, [DT(C)/DS(C)]. **(A)** Venn diagram showing common and specific DATs in DT(D/C), DS(D/C) and DT(C)/DS(C) comparisons, where total number of transcripts are shown by black color font, and up- and down-regulated transcripts are presented by red and blue color fonts, respectively. **(B, C)** Bubble plots showing significantly enriched pathways among up- **(B)** and down-regulated **(C)** transcripts in DT(D/C), DS(D/C) and DT(C)/DS(C) comparisons. Top 20 pathways with *P*-value ≤ 0.05 were selected. The bubble size defines the number of transcripts assigned to the pathway and bubble color represents *P*-value as per the given scale.

RNA-sequencing of root samples from the two chickpea genotypes, ICC 4958 (DT) and ICC 1882 (DS) was performed under control (C) and drought stress (D) conditions. The high-quality filtered reads for each sample were mapped to the chickpea genome (**Table S1**). The reference-guided assembly resulted in a total of 30978 gene loci, including 27881 known and 3097 novel loci (**Table S2**). Differential expression analysis revealed a total of 1718 (478 up- and 1240 down-regulated) and 1702 (680 up- and 1022 down-regulated) Differentially Abundant Transcripts (DATs) in DT [DT(D/C)] and DS [DS(D/C)] genotypes, respectively, under drought stress. Further, at least 1222 (864 up- and 358 down-regulated) DATs were identified between DT and DS genotypes under control conditions [DT(C)/DS(C)] (**Figures 1**, **S1A**) (**Table S3**). A comparative analysis of DT(D/C), DS(D/C) and DT(C)/DS(C) identified common and specific DATs among different comparisons. A total of 1128 (409 up- and 719 down-regulated), 841 (406 up- and 435 down-regulated), and 900 (644 up- and 256 down-regulated) DATs were specific for DT(D/C), DS(D/C) and DT(C)/DS(C) comparisons, respectively. However, 580 (62 up- and 518 down-regulated) DATs were common between DT(D/C) and DS(D/C), and only 31 (1 up- and 30 down-regulated) DATs were common among all the comparisons (**Figure 1A**).

To investigate the metabolic pathways associated with DATs, pathway enrichment analysis was performed for the up- or down-regulated sets of genes for DT(D/C), DS(D/C) and DT(C)/DS(C). A total of 136, 108 and 174 significantly enriched pathways were identified among the up-regulated genes in DT(D/C), DS(D/C) and DT(C)/DS(C), respectively (**Figure 1B**). The most enriched pathways for DT(D/C) included plant hormone signaling, phenylpropanoid biosynthesis, carbon metabolism, anthocyanin modification, sucrose biosynthesis, glycolysis, gluconeogenesis, galactose metabolism, pentose phosphate pathway, and fructose and mannose metabolism. Among the up-regulated genes in DS(D/C), those encoding ABC transporters, and involved in callose biosynthesis, and circadian rhythm were enriched. However, DT(C)/DS(C) showed enrichment of plant-pathogen interaction, cellulose biosynthesis, starch and sucrose metabolism, phospholipases, phospholipid remodeling, wnt signaling pathway, triacylglycerol biosynthesis/degradation, glycerolipid metabolism, phosphatidylcholine acyl editing and MAPK signaling pathways among the up-regulated genes. The nitrogen metabolism pathway was enriched in both DT(D/C) and DS(D/C), while the diterpenoid biosynthesis pathway was enriched among the up-regulated genes in all the comparisons. Similarly, down-regulated genes in DT(D/C), DS(D/C) and DT(C)/DS(C) were found to be involved in 224, 209 and 91 pathways, respectively (**Figure 1C**). Most enriched pathways among down-regulated genes in DT(D/C) were, triacylglycerol biosynthesis and degradation, phospholipases, and cysteine and methionine metabolism, whereas terpenoid biosynthesis, flavonoid biosynthesis, glycolysis/gluconeogenesis, coumarins biosynthesis, amino sugar and nucleotide sugar metabolism, and fatty acid degradation pathways were enriched in DS(D/C). The pathways related to plant-pathogen interaction, MAPK signaling pathway, phenylpropanoid biosynthesis, pyridine nucleotide cycling, phosphatidylcholine acyl editing, and cellulose biosynthesis were enriched in both DT(D/C) and DS(D/C), whereas plant hormone signal transduction pathway was enriched in DT(D/C), DS(D/C) and DT(C)/DS(C).

To further investigate the biological functions of DATs, comparative GO enrichment analysis of the up- (**Figure S1B**) or down-regulated (**Figure S1C**) genes in DT(D/C), DS(D/C) and DT(C)/DS(C) comparisons were performed. The up-regulated genes in DT(D/C) were involved mainly in amino acid metabolism and transport, ion transport, response to ions, and cell wall modification. However, negative regulation of biosynthesis and development, pigment metabolism, terpene metabolism, DNA modifications, regulation of transport, DNA maintenance and recombination, and hormone stimulus biological process terms were enriched in DS(D/C). GO terms related to signaling, root morphogenesis, hormone signaling, phenylpropanoid metabolism, and carbohydrate metabolism were enriched in DT(C)/DS(C). GO terms, including gene silencing, cell division, DNA replication and repair, and root development were represented in both DS(D/C) and DT(C)/DS(C). The down-regulated genes were involved in the signaling pathway, response to abiotic stress, immune response, hormone signaling, carbohydrate biosynthesis and protein modification in DT(D/C). Lipid biosynthesis and cellular respiration terms were enriched in DS(D/C) and DT(C)/DS(C), respectively. However, hormone stimulus, response to biotic and abiotic stimulus, and cell wall modification were commonly represented in both DS(D/C) and DT(C)/DS(C).

### 3.2 Differentially abundant proteins and pathway/GO enrichment analysis

**Figure 2:**
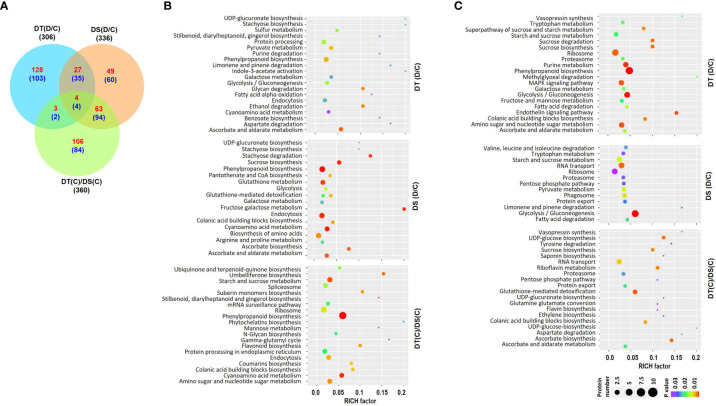
Differentially abundant proteins (DAPs) in drought-tolerant (DT) and drought-sensitive (DS) chickpea genotypes under control (C) and drought stress (D) conditions. DAPs in the DT and DS genotypes were identified under drought stress as compared to control, DT(D/C) and DS(D/C), respectively, and between DT and DS genotypes under control condition, [DT(C)/DS(C)]. **(A)** Venn diagram showing common and specific DAPs in DT(D/C), DS(D/C) and DT(C)/DS(C) comparisons, where total number of proteins are shown by black color font, and up- and down-regulated proteins are presented by red and blue color fonts, respectively. **(B, C)** Bubble plots showing significantly enriched pathways among up- **(B)** and down-regulated **(C)** proteins in DT(D/C), DS(D/C) and DT(C)/DS(C) comparisons. Top 20 pathways with *P*-value ≤ 0.05 were selected. The bubble size defines the number of proteins assigned to the pathway and bubble color represents *P*-value as per the given scale.

Overall proteome analysis identified a total of 25742 peptides representing 2430 unique proteins in all the samples. Among these, 2180, 2205, 2171 and 2138 proteins were identified in DT(C), DT(D), DS(C) and DS(D) samples, respectively (**Table S4**). The differential expression analysis revealed a total of 306 (162 up- and 144 down-regulated), 336 (143 up- and 193 down-regulated) and 360 (176 up- and 184 down-regulated) DAPs in the DT(D/C), DS(D/C) and DT(C)/DS(C) comparisons, respectively (**Figures 2A**, **S2A**) (**Table S5**). A comparative analysis of DT(D/C), DS(D/C) and DT(C)/DS(C) identified 231 (128 up- and 103 down-regulated), 109 (49 up- and 60 down-regulated) and 190 (106 up- and 84 down-regulated) DAPs specific to DT(D/C), DS(D/C), DT(C)/DS(C), respectively. However, 70 (31 up- and 39 down-regulated) DAPs were common between DT(D/C) and DS(D/C), and 8 (4 up- and 4 down-regulated) DAPs were common among all the comparisons (**Figure 2A**).

The pathway enrichment analysis was performed for the up- or down-regulated proteins in DT(D/C), DS(D/C) and DT(C)/DS(C) comparisons. The up-regulated proteins contributed to 116, 128 and 14 metabolic pathways, whereas down-regulated proteins were involved in 115, 56 and 83 metabolic pathways in DT(D/C), DS(D/C) and DT(C)/DS(C), respectively. The up-regulated proteins were involved mainly in pyruvate metabolism and protein processing in DT(D/C), amino acid biosynthesis, sucrose biosynthesis and glutathione metabolism in DS(D/C), and starch and sucrose metabolism, amino sugar and nucleotide sugar metabolism, ribosome, spliceosome and protein processing in DT(C)/DS(C) (**Figure 2B**). The galactose metabolism, glycolysis/gluconeogenesis, and ascorbate and aldarate metabolism pathways were commonly enriched in DT(D/C) and DS(D/C), whereas phenylpropanoid biosynthesis, endocytosis and cyanoamino acid metabolism were detected in all the three comparisons, DT(D/C), DS(D/C) and DT(C)/DS(C) (**Figure 2B**). Similarly, enriched pathways among the down-regulated proteins were, purine metabolism, amino sugar and nucleotide sugar metabolism, MAPK signaling pathway and phenylpropanoid biosynthesis in DT(D/C); RNA transport, phagosome and pyruvate metabolism in DS(D/C); and glutathione-mediated detoxification and RNA transport in DT(C)/DS(C) (**Figure 2C**). Glycolysis/gluconeogenesis, ribosome, proteasome, fatty acid degradation, and starch and sucrose metabolism were enriched in both DT(D/C) and DS(D/C), whereas sucrose biosynthesis, colanic acid building blocks biosynthesis, and ascorbate and aldarate metabolism were common in DT(D/C) and DT(C)/DS(C) (**Figure 2C**).

Next, GO enrichment analyses of up- or down-regulated proteins in DT(D/C), DS(D/C) and DT(C)/DS(C) were performed to gain more insights into their biological functions (**Figures S2B, C**). The up-regulated proteins in DT(D/C) were enriched in fatty acid metabolism, auxin homeostasis and cell growth related GO terms. However, in DS(D/C) transport and, purine and pyrimidine metabolism; and in DT(C)/DS(C), hormone signaling, amino acid metabolic process, cell wall modification, chromosome organization and phenylpropanoid pathway were enriched. The amino acid metabolism and MAPK signaling were common in DS(D/C) and DT(C)/DS(C). The GO enrichment analysis of down-regulated proteins suggested the involvement of phenylpropanoid metabolism, jasmonic acid and ethylene dependent signaling, abiotic stress, immune response and nucleotide metabolism in DT(D/C). However, hormone signaling, RNA splicing, fatty acid oxidation, protein targeting, transport and localization terms were specific to DT(C)/DS(C).

### 3.3 Differential metabolite profiling and pathway enrichment analysis

**Figure 3:**
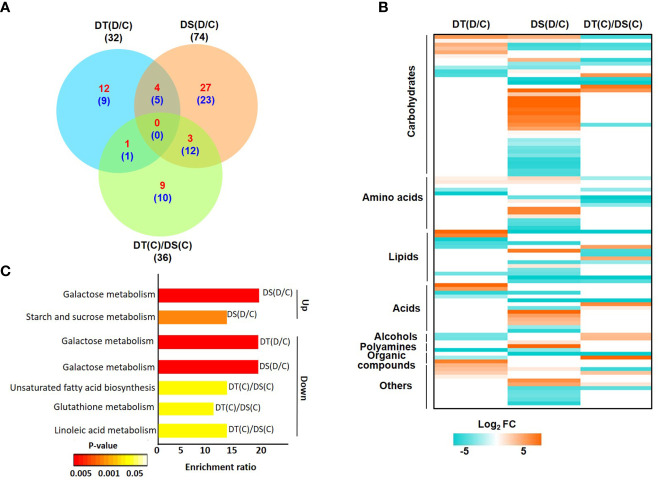
Differentially abundant metabolites (DAMs) in drought-tolerant (DT) and drought-sensitive (DS) chickpea genotypes under control (C) and drought stress (D) conditions. DAMs in the DT and DS genotypes were identified under drought stress as compared to control, DT(D/C) and DS(D/C) respectively, and between DT and DS genotypes under control condition, [DT(C)/DS(C)]. **(A)** Venn diagram showing common and specific DAMs in DT(D/C), DS(D/C) and DT(C)/DS(C) comparisons, where total number of metabolites are shown by black color font, and up- and down-regulated metabolites are presented by red and blue color fonts, respectively. **(B)** Heatmap of DAMs identified in DT(D/C), DS(D/C) and DT(C)/DS(C) comparisons. DAMs with log_2_ fold change (FC) of ≥1 (up-regulated) or ≤-1 (down-regulated) at *P*-value ≤ 0.05 are shown. Various classes of metabolites represented among the DAMs are given. The name of each metabolite along with fold change value is given in **Figure S3C**. Scale at the bottom represents log_2_ FC. **(C)** Enrichment ratio of the significantly enriched metabolic pathways represented among up- and down-regulated metabolites for DT(D/C), DS(D/C) and DT(C)/DS(C) comparisons are shown.

After processing the raw data obtained *via* GC-MS analysis, we identified high-confidence metabolites in all the samples. Overall, a total of 133 high-confidence metabolites were obtained in all the samples, of which 87, 98, 94 and 88 metabolites were represented in DT(C), DT(D), DS(C) and DS(D), respectively (**Figure S3A**; **Table S6**). The analysis identified a total of 32 (17 up- and 15 down-regulated), 74 (34 up- and 40 down-regulated) and 36 (13 up- and 23 down-regulated) DAMs in DT(D/C), DS(D/C) and DT(C)/DS(C) comparisons, respectively (**Figure 3A**; **Table S7**). A comparative analysis of DT(D/C), DS(D/C) and DT(C)/DS(C) identified 21 (12 up- and 9 down-regulated), 50 (27 up- and 23 down-regulated) and 19 (9 up- and 10 down-regulated) DAMs specific to DT(D/C), DS(D/C) and DT(C)/DS(C), respectively. However, 9 DAMs (4 up- and 5 down-regulated) were common between DT(D/C) and DS(D/C) (**Figure 3A**). The DAMs identified in all three comparisons, DT(D/C), DS(D/C) and DT(C)/DS(C), were comprised of different metabolite classes (**Figure 3B**). Among these, the metabolites representing carbohydrates, lipids and amino acids were most abundant in all the three comparisons. However, metabolites representing acids, alcohols, polyamines, organic compounds and other classes were comparatively less prominent (**Figures S3B, C**).

The pathway enrichment analysis of DAMs revealed that up-regulated metabolites in DT(D/C) were majorly involved in the arginine biosynthesis pathway (**Figure S4**). DAMs in DS(D/C) represented galactose metabolism, starch and sucrose metabolism, fructose and mannose metabolism as the most enriched pathways. However, glycine, serine and threonine metabolism, and aminoacyl-tRNA biosynthesis pathways were common in DT(D/C) and DS(D/C). The down-regulated metabolites were involved in glyoxylate and dicarboxylate metabolism, butanoate metabolism, alanine, aspartate and glutamate metabolism in DT(D/C), fructose and mannose metabolism in DS(D/C), and biosynthesis of unsaturated fatty acids, cyanoamino acid metabolism and linoleic acid metabolism in DT(C)/DS(C). Furthermore, galactose metabolism pathway was common between DT(D/C) and DS(D/C), glutathione metabolism between DT(D/C) and DT(C)/DS(C), and aminoacyl-tRNA biosynthesis between DS(D/C) and DT(C)/DS(C) (**Figure S4**). Among the identified pathways, galactose metabolism, and starch and sucrose metabolism were most enriched among the up-regulated metabolites in DS(D/C). However, down-regulated metabolites showed enrichment of galactose metabolism in DT(D/C) and DS(D/C), and unsaturated fatty acid biosynthesis, glutathione metabolism and linoleic acid metabolism in DT(C)/DS(C) (**Figure 3C**).

### 3.4 Co-expression network analysis of genes, proteins and metabolites

Co-expression analysis was performed *via* WGCNA to investigate the co-expressed transcripts, proteins and metabolites among DT(C), DT(D), DS(C) and DS(D) samples. The WGCNA is a co-expression network analysis utilized for the analyses of gene expression data providing the correlation of coexpressed genes with the samples (Langfelder and Horvath, 2008). For the transcriptome data, hierarchical clustering of 9847 genes (with variance > 0.1) *via* WGCNA generated 30 co-expressed modules. These modules were further clustered according to dissimilarity measure (1-TOM) and 11 highly co-expressed modules were obtained (**Figure S5A**). The module size varied from 60 (steelblue) to 2252 (black) genes. Further, the co-expressed modules were correlated with the samples (**Figure S6A**), and the modules with correlation coefficient (r) of ≥ 0.50 were assigned to the respective tissue sample. In total, five [brown (r = 0.98), grey60 (r = 0.83), midnightblue (r = 0.75), darkorange (r = 0.64) and blue (r = 0.54)] modules were found correlated with DT(C) and four [cyan (r = 0.78), black (r = 0.54), white (r = 0.95) and orange (r = 0.64)] modules with DT(D). One module, blue (r = 0.68) and steelblue (r = 0.53) was correlated with each of DS(C) and DS(D), respectively.

Similarly, WGCNA analysis for 2430 proteins generated 9 co-expressed modules (**Figure S5B**). The number of proteins identified in the modules ranged from 34 (darkgrey) to 551 (blue). The co-expressed modules showing correlation coefficient (r) of ≥ 0.35 with their respective samples were selected. The darkturquoise (r = 0.36) module showing correlation with DT(C); blue (r = 0.99), grey60 (r = 0.65) and darkturquoise (r = 0.37) with DT(D); darkgreen (r = 0.52), royalblue (r = 0.92), green (r = 0.49) and brown (r = 0.82) with DS(C); and darkgrey (r = 0.53) and royalblue (r = 0.85) with DS(D) were selected for further analysis (**Figure S6B**). The WGCNA analysis for 133 metabolites generated 5 modules comprised of 15 (green) to 48 (darkturquoise) metabolites. The co-expressed modules, highly correlated (r ≥ 0.50) with the samples, including darkturquoise (r = 0.74) module for DT(C); brown (r = 0.67) and darkturquoise (r = 0.82) for DT(D); yellow (r = 0.75) and darkturquoise (r = 0.97) for DS(C); and blue (r = 0.53) and green (r = 0.88) for DS(D), were identified (**Figure S6C**).

### 3.5 Integrated transcriptome, proteome and metabolome analysis

**Figure 4:**
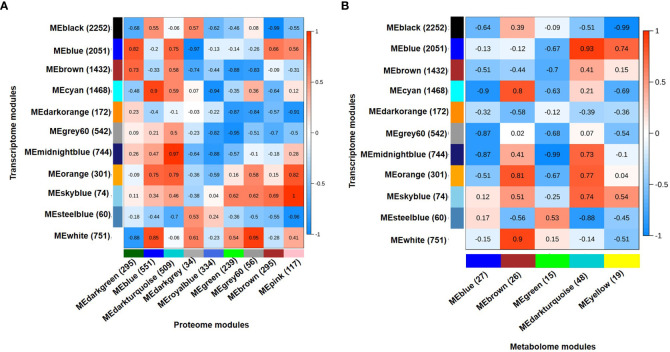
Integrated analysis for transcriptome, proteome and metabolome using weighted gene co-expression network analysis (WGCNA). **(A)** Integration of transcriptome and proteome data using eigenvector for co-expressed gene modules (eigengenes) and co-expressed protein modules (eigenproteins). A total of 11 eigengenes and 9 eigenproteins generated a matrix showing correlated eigengenes-eigenproteins. Highly correlated modules were selected at correlation coefficients (r) ≥ 0.5. **(B)** Integration of transcriptome and metabolome data using eigenvector for co-expressed gene modules (eigengenes) and co-expressed metabolite modules (eigenmetabolites). A total of 11 eigengene and 5 eigenmetabolite generated a matrix showing correlated eigengenes-eigenmetabolites. Highly correlated modules were selected at correlation coefficients (r) ≥ 0.35. Correlation coefficient (r) for each module pair is shown as per scales shown on the right side. The total number of genes, proteins and metabolites included in each module are shown in parenthesis.

Integration of transcriptome, proteome and metabolome data was performed using module eigenvalues obtained for each data individually *via* WGCNA analysis. The module eigenvalue represents the first principal component and reflects the expression profiles of the given module. The integration of 11 co-expressed gene modules (eigengenes) and 9 co-expressed protein modules (eigenproteins) revealed five highly correlated eigengene-eigenprotein modules (**Figure 4A**). Similarly, 11 co-expressed gene modules (eigengenes) and 5 co-expressed metabolite modules (eigenmetabolites) identified highly correlated eigengene-eigenmetabolite modules (**Figure 4B**). The transcriptome modules correlated with both proteome and metabolome modules were selected for further analysis. Therefore, three transcriptome (mignightblue, brown and blue), one proteome (darkturquoise) and one metabolome (darkturquoise) modules were found correlated with DT(C), whereas four transcriptome (cyan, black, white and orange), three proteome (blue, darkturquoise and grey60) and two metabolome (brown and darkturquoise) modules were associated with DT(D). Similarly, one transcriptome (blue), two proteome (brown and darkgreen), and two metabolome (yellow and darkturquoise) modules were selected for DS(C). However, one module each of transcriptome (steelblue), proteome (darkgrey) and metabolome (green) was correlated with DS(D). These selected modules comprising of co-expressed genes, proteins and metabolites were analyzed together to identify the represented metabolic pathways and transcription factors among them. The stress-responsive pathways, including phosphatidylinositol (PI) signaling, glutathione metabolism and glycolysis/gluconeogenesis were found to be enriched and investigated further in more detail along with transcription factors to reveal the regulation of genes, proteins and metabolites involved.

#### 3.5.1 Phosphatidylinositol signaling pathway

**Figure 5:**
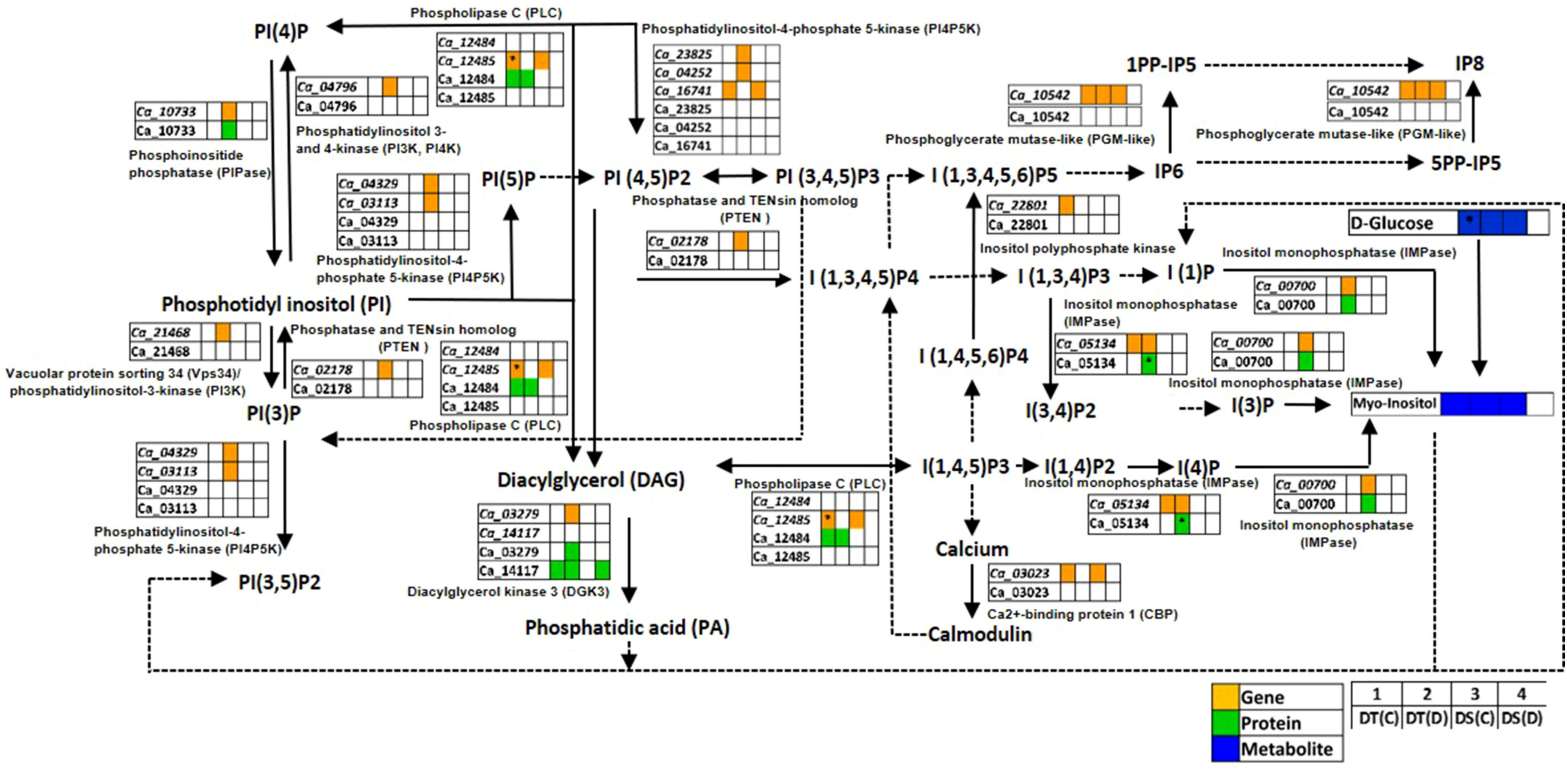
Phosphatidylinositol (PI) signaling pathway showing co-expressed genes, proteins and metabolites in drought-tolerant (DT) and drought-sensitive (DS) chickpea genotypes under control (C) and drought (D) conditions. Co-expressed genes, proteins and metabolites are shown by orange, green and blue colored boxes, respectively. The up-regulated genes, proteins and metabolites are marked (*). PP-IPs- Diphosphoinositol phosphates; PI- Phosphatidylinositol; PIP, Phosphatidylinositol phosphate; IP, Inositol phosphate. Down-regulated genes, proteins and metabolites were not reported in this pathway.

PI signaling related membrane lipids, phosphatidylinositols (PIs), phosphatidylinositol phosphates (PIPs) and inositol phosphates (IPs) play a significant role in plant adaptation to abiotic stress (Heilmann, 2016). We found genes and proteins related to the PI signaling pathway to be co-expressed in one or more of the conditions analyzed (**Figure 5**; **Table S8**). Notably, the genes encoding inositol monophosphatase (IMPase; *Ca_00700*), diacylglycerol kinase 3 (DGK3; *Ca_03279*), phosphoinositide phosphatase (PIPase; *Ca_10733*), phosphatidylinositol-4-phosphate 5-kinase (PI4P5K; *Ca_03113*, PI4P5K1; *Ca_04252*), inositol-4-phosphate 5-kinase2 (I4P5K2; *Ca_23825*), vacuolar protein sorting 34 (Vps34)/phosphatidylinositol-3-kinase (PI3K; *Ca_21468*), phosphatidylinositol 3- and 4-kinase (PI3K, PI4K; *Ca_04796*), phosphatidylinositol 3-phosphate 5-kinase (PI3P5K; *Ca_04329*), and phosphatase and TENsin homolog 2 (PTEN 2; *Ca_02178*) were co-expressed specifically in DT(D). Furthermore, IMPase (Ca_00700 and Ca_05134), DGK3 (Ca_03279) and PIPase (Ca_10733) proteins were also found to be co-expressed in DT(D). The IMPase protein (Ca_05134) was up-regulated in DT(D/C). Moreover, an enhanced level of myo-inositol (an osmoprotectant) was detected in DT(C), DT(D) and DS(C). These co-expressed/stress-responsive genes, proteins and metabolites might contribute to coordinated regulation of PI signaling to impart better drought adaptation in the DT genotype.

#### 3.5.2 Glutathione metabolism pathway

**Figure 6:**
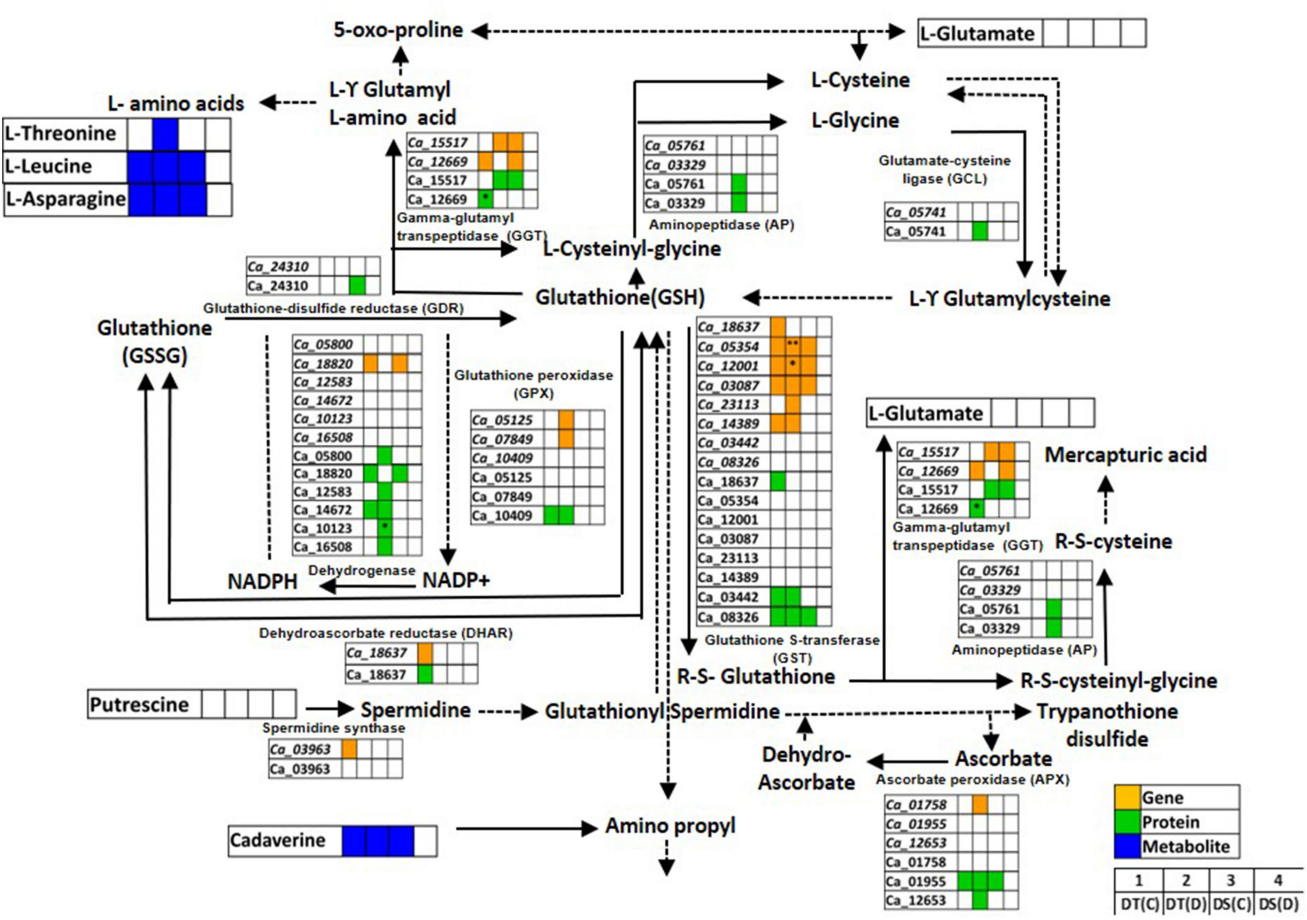
Glutathione metabolism pathway showing co-expressed genes, proteins and metabolites in drought-tolerant (DT) and drought-sensitive (DS) chickpea genotypes under control (C) and drought (D) conditions. Co-expressed genes, proteins and metabolites are shown by orange, green and blue colored boxes, respectively. Up- (*) and down-regulated (**) genes, proteins and metabolites are marked. GSH- Glutathione reduced; GSSG- Glutathione oxidized.

Glutathione metabolism is an antioxidant defense system that prevents cellular damage during abiotic stress (Banerjee and Roychoudhury, 2019). We identified genes, proteins and metabolites related to glutathione metabolism co-expressed in DT(C), DT(D), DS(C) and DS(D) samples (**Figure 6**; **Table S9**). The genes of glutathione S-transferase classes, namely, lambda (GST; *Ca_12001*) and TAU (GSTU25; *Ca_03087*, GSTU7; *Ca_05354*), and ascorbate peroxidase 3 (APX3; *Ca_01955*), and protein, GSTU8 (Ca_08326) were found to be co-expressed in DT(C), DT(D) and DS(C). The co-expression of these genes and proteins in both DT and DS genotypes may represent their fundamental role in the glutathione metabolism (Moons, 2005). However, genotype-specific co-expression was observed for GSTU19 (*Ca_14389*) gene, and GST7 (Ca_03442), glutathione peroxidase 3 (GPX3; Ca_10409) and glucose-6-phosphate dehydrogenase 6 (G6PDH; Ca_14672) proteins in DT(C) and DT(D). Importantly, correlation of genes including, GPX4 (*Ca_07849)*, APX6 (*Ca_01758)*, cysteine peroxiredoxin 1 (CysPrx1; *Ca_05125)*, GSTU20 (*Ca_23113)*, and proteins, 6-phosphogluconate dehydrogenase (6PGDH; Ca_05800, *Ca_16508*), cytosol aminopeptidase (AP; Ca_03329), peptidase M1 (PepM1; Ca_05761), isocitrate/isopropyl malate dehydrogenase (IDH; Ca_12583), thylakoidal ascorbate peroxidase (tAPX; Ca_12653), glutamate-cysteine ligase (GCL; Ca_05741) and G6PDH1 (Ca_10123) specifically in DT(D) indicated their important role in drought stress. Furthermore, genes of glutathione S-transferase classes, GSTU7 (*Ca_05354*) and GST (*Ca_12001*), and G6PDH1 (*Ca*_10123) protein were differentially abundant in DT(D/C). L-threonine was co-expressed mainly in DT(D), whereas cadaverine, leucine and asparagine were co-expressed in DT(C), DT(D) and DS(C). The co-expressed/stress-responsive genes, proteins and metabolites may confer an improved glutathione metabolism in the DT genotype.

#### 3.5.3 Glycolysis/gluconeogenesis pathway

**Figure 7:**
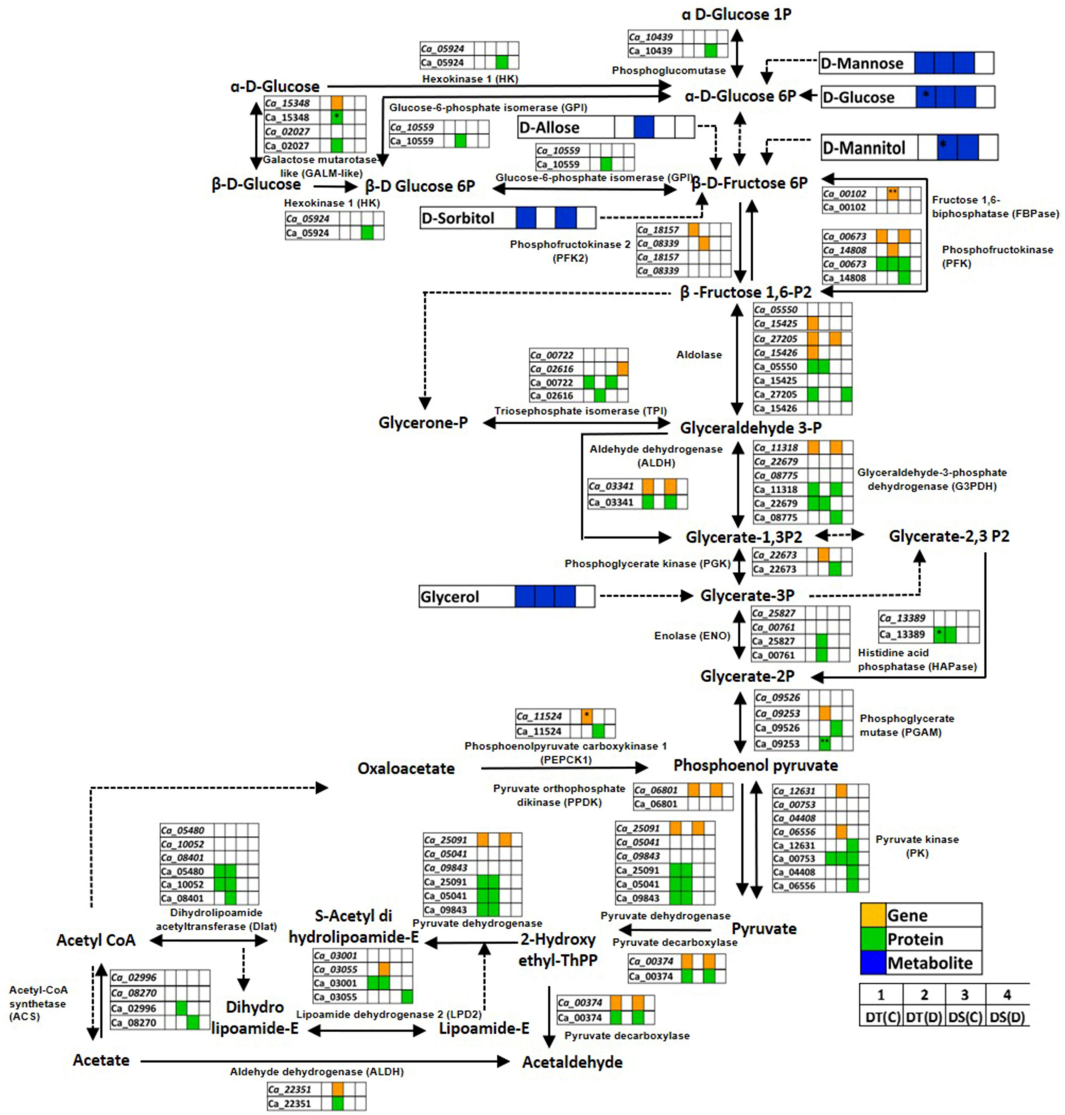
Glycolysis/gluconeogenesis pathway showing co-expressed genes, proteins and metabolites in drought-tolerant (DT) and drought-sensitive (DS) chickpea genotypes under control (C) and drought (D) conditions. Co-expressed genes, proteins and metabolites are shown by orange, green and blue colored boxes, respectively. Up- (*) and down-regulated (**) genes, proteins and metabolites are marked.

Glycolysis/gluconeogenesis is a fundamental carbohydrate metabolism pathway regulating cellular carbon metabolism and energy requirement. We found several glycolysis/gluconeogenesis associated genes, proteins and metabolites to be co-expressed in DT(C), DT(D), DS(C) and DS(D) (**Figure 7**; **Table S10**). The genes, namely aldolase (*Ca_27205*), glyceraldehyde-3-phosphate dehydrogenase C2 (GAPDHC2; *Ca_11318*), pyruvate decarboxylase-2 (PDC2; *Ca_00374*), pyruvate dehydrogenase (PDH) complex E1 alpha subunit (*Ca_25091*), phosphofructokinase (PFK; *Ca_00673*), aldehyde dehydrogenase 11A3 (ALDH11A3; Ca_03341), pyruvate orthophosphate dikinase (PPDK) (*Ca_06801*), and proteins encoding GAPDHC2 (Ca_11318), PDC2 (Ca_00374), PFK (Ca_00673), ALDH11A3 (Ca_03341), triosephosphate isomerase (TPI; Ca_00722) and pyruvate kinase (PK) (Ca_00753) were co-expressed in both DT(C) and DS(C). These co-expressed genes and proteins were considered key regulators of glycolysis/gluconeogenesis. However, PK (*Ca_12631*, *Ca_06556*), galactose mutarotase-like (GALM-like; *Ca_02027*, *Ca_15348*), PFK (*Ca_14808*), PFK4 (*Ca_08339*), phosphoglycerate kinase (PGK; *Ca_22673*), phosphoenolpyruvate carboxykinase 1 (PEPCK1; *Ca_11524*), lipoamide dehydrogenase 2 (LPD2; *Ca_03055*), ALDH7B4 (*Ca_22351*), phosphoglycerate mutase (PGAM; *Ca_09253*) and fructose 1,6-biphosphatase (FBPase; *Ca_00102*) genes, and GALM-like (Ca_15348); ALDH7B4 (Ca_22351); PGAM (Ca_09253), TPI (Ca_02616), enolase (ENO; Ca_00761), ENO1 (Ca_25827), glucose-6-phosphate isomerase (GPI; Ca_10559), dihydrolipoamide acetyltransferase (Dlat; Ca_08401) and acyl-activating enzyme 7 (AAE7; Ca_02996) proteins were correlated mainly with DT(D). Notably, FBPase (*Ca_00102*), PEPCK1 (*Ca_11524*), GALM-like (Ca_15348) and PGAM (Ca_09253) were differentially abundant in DT(D/C). The metabolites, including D-glucose, D-mannose and glycerol were co-expressed in DT(C), DT(D) and DS(C). However, D-mannitol was co-expressed mainly in DT(D) and DS(C); sorbitol in DT(C) and DS(C), and D-allose in DT(D). The up-regulation of D-glucose and D-mannitol was observed in DT(C)/DS(C) and DT(D/C), respectively. These co-expressed/stress-responsive genes, proteins and metabolites in the glycolysis/gluconeogenesis pathway possibly contribute to drought stress adaptation in the DT genotype.

### 3.6 Transcription factor encoding genes

TF encoding genes represented among the co-expressed genes of DT(C), DT(D), DS(C) and DS(D) were investigated. In total, 735 TFs (belonging to 79 families), including 319, 267, 143 and 6 were identified in DT(C), DT(D), DS(C) and DS(D), respectively (**Figure S7A**). The members of TF families, including MYB (55, 7.51%), bHLH (55, 7.51%), AP2-EREBP (45, 6.14%), HB (38, 5.2%), C3H (28, 3.8%), MADS, (26, 3.55%), bZIP (26, 3.55%), WRKY (26, 3.55%) and NAC (24, 3.27%) were most represented (**Figure S7B**). MYB, bHLH, HB and AP2-EREBP were the most abundant TF families identified in DT(C) and DT(D). A total of 39 TFs (14 up- and 25 down-regulated) were identified in black, cyan, orange and white modules correlated with DT(D). The DT(C) correlated modules (blue, brown, midnight blue and grey60) harbored 28 differentially abundant TFs (18 up- and 10 down-regulated). These modules represented the members of several TF-families (GNAT, GRAS, C3H, HB, G2-like, bZIP, PLATZ, sigma70-like, CCAAT, WRKY, Aux/IAA and C2C2-Dof) that are well known to be involved in the regulation of drought stress response (**Figure S7C**).

### 3.7 Identification of *QTL-hotspot* associated candidate genes/proteins involved in drought stress

**Figure 8:**
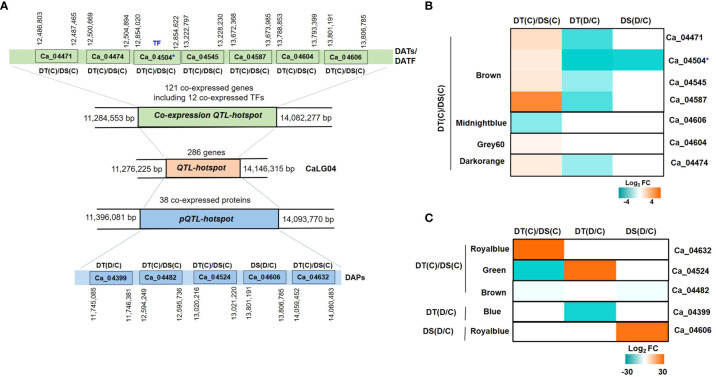
Identification of quantitative trait loci-hotspot (*QTL*-*hotspot*) associated drought responsive genes, proteins and transcription factors (TFs) in drought-tolerant (DT) and drought-sensitive (DS) chickpea genotypes under control (C) and drought (D) conditions. **(A)** *QTL*-*hotspot* region spanning 3Mb (Ca4_11,276,225 to Ca4_14,146,315 bp) harboring several QTLs for drought tolerance related traits in linkage group 04 (CaLG04) in chickpea. *QTL-hotspot* region (11, 284,553 bp to 14,082,277 bp) comprised of co-expressed genes is considered as co-expression *QTL-hotspot* region. *QTL*-*hotspot* region (11,396,081 to 14,093,770 bp) comprised of co-expressed proteins is considered as protein quantitative trait loci-hotspot (*pQTL-hotspot*). Differentially abundant transcripts (DATs) and differentially abundant proteins (DAPs) of co-expression *QTL-hotspot* and *pQTL-hotspot*, respectively, are shown with their co-ordinates. **(B)** Heatmap of co-expression *QTL-hotspot* region associated DATs and DA-TFs identified in different modules of DT(C)/DS(C). **(C)** Heatmap of *pQTL*-*hotspot* region associated DAPs identified in different modules of DT(C)/DS(C), DT(D/C) and DS(D/C). * denotes differentially abundant TF.

A *QTL-hotspot* region spanning 3 Mb (Ca4_11,276,225 to Ca4_14,146,315 bp) harboring several QTLs for drought tolerance related traits has been identified in linkage group 4 (CaLG04) in chickpea (Varshney et al., 2014). We identified a total of 286 annotated protein-coding genes located within the *QTL*-*hotspot* region (**Figure 8A**). The *QTL*-*hotspot* region (11,284,553 bp to 14,082,277 bp) comprised of co-expressed genes was considered as co-expression *QTL-hotspot* (**Figure 8A**). The co-expression *QTL-hotspot* contained a total of 121 co-expressed genes. Among these, 57 genes (blue, brown, midnightblue, grey60 and darkorange modules) in DT(C), 42 genes (black, cyan, orange and white modules) in DT(D) and 22 genes (blue module) in DS(C) were co-expressed (**Figure S8A**). Twenty genes in blue module were found to be common between DT(C) and DS(C), however no genes were co-expressed in DS(D) and DT(D). Notably, at least 12 co-expressed genes encoded TFs, such as MADS-box (*Ca_04518*), OFP (*Ca_04584*), RWP-RK (*Ca_04456*), and AP2-EREBP (*Ca_04503*, *Ca_04504*) were specific to DT(C), Alfin-like (*Ca_04596*), SNF2 (*Ca_04622*), HSF (*Ca_04488*) and MYB (*Ca_04621*) were present in DT(D), whereas Trihelix (*Ca_04385, Ca_04387*) and AP2-EREBP (*Ca_04370*) were co-expressed in both DT(C) and DS(C) (**Figure S8B**). Further, *QTL*-*hotspot* region (11,396,081 to 14,093,770 bp) comprised of 38 co-expressed proteins was considered as protein *QTL-hotspot* (*pQTL-hotspot*) (**Figure 8A**). The *pQTL*-*hotspot* region associated proteins were present in darkturquoise module of DT(C); darkturquoise, grey60 and blue of DT(D); royalblue, green, darkgreen and brown of DS(C) and, royalblue and darkgrey modules of DS(D) (**Figure S8C**).

We further investigated the differential abundance behavior of *QTL-hotspot* associated co-expressed genes and proteins. In total, seven (6 up- and 1 down-regulated) DATs including one TF in DT(C)/DS(C) were identified within the co-expression *QTL-hotspot*. The genes encoding receptor like protein 21 (RLP21; *Ca_04471*), stabilizer of iron transporter SufD/polynucleotidyl transferase (PNT; *Ca_04545*), cytochrome P450 family 76 (cyt 450; *Ca_04587*), copper amine oxidase (CuAOs; *Ca_04604*) and myosin heavy chain-related protein (*Ca_04474*) were up-regulated, whereas PLAT/LH2 domain-containing lipoxygenase (*Ca_04606*) was down-regulated in DT(C)/DS(C) (**Figure 8B**). In addition, one co-expression *QTL*-*hotspot* region associated gene encoding dehydration-responsive element binding (DREB; *Ca_04504*) TF was up-regulated in DT(C)/DS(C) (**Figure 8B**). Among the *pQTL-hotspot* region associated co-expressed proteins, nucleic acid binding protein (Ca_04399) was down-regulated in DT(D/C), whereas PLAT/LH2 domain-containing lipoxygenase family protein (Ca_04606) was up-regulated in DS(D/C). However, oligosaccharyltransferase (OST) complex/magnesium transporter family protein (Ca_04632) was up-regulated, and carboxylesterase 20 (Ca_04524) and membrane-associated progesterone binding protein 3 (Ca_04482) were downregulated in DT(C)/DS(C) (**Figure 8C**). Overall, the co-expressed/up-regulated genes, proteins and/or TFs located within the *QTL*-*hotspot* region represent important candidates for drought tolerance in chickpea.

### 3.8 Validation of differential abundance of selected genes *via* RT-qPCR

The differential abundance of selected candidate genes in different comparisons was determined by RT-qPCR. A total of 17 selected genes involved in PI signaling, glutathione metabolism and, glycolysis/gluconeogenesis pathway, and those located in *QTL-hotspot* and TF related genes that exhibited differential abundance in RNA-seq data were analyzed by RT-qPCR analysis. The expression analysis of the genes revealed a high correlation (Pearson correlation, 0.76) between the results obtained from RT-qPCR and RNA-seq (**Figure S9**).

## 4 Discussion

Drought stress significantly affects chickpea growth and productivity *via* altering the underlying molecular mechanism/response (Nayyar et al., 2006). An integrated multi-omics analysis can elucidate important biological processes, metabolic pathways and regulatory networks in plants (Amiour et al., 2012; Srivastava et al., 2013; Sudre et al., 2013; Zeng et al., 2013 Remmers et al., 2018; Li et al., 2019b; Wang et al., 2019; Yun et al., 2019; Chin et al., 2020; Moreno et al., 2021; Bittencourt et al., 2022; Leão et al., 2022; Shu et al., 2022). However, such integrated analysis to decipher plant response to abiotic stress is lacking in chickpea. In this study, we performed transcriptomics, proteomics and metabolomics analyses of two well-characterized genotypes with contrasting drought response/tolerance (ICC 4958 as DT and ICC 1882 as DS) to gain insights into the molecular mechanisms underlying drought stress in chickpea. Further, integration of multi-omics data was performed to provide a better understanding of drought tolerance in chickpea.

### 4.1 DATs, DAPs and DAMs participate in specific metabolic pathways/processes

Plant adaptation to abiotic stress is mainly initiated by stress-responsive genes and/or proteins (Kosova et al., 2018; Nawae et al., 2020; Kosova et al., 2021; Kausar et al., 2022). We investigated the differential abundance of genes, proteins and metabolites in DT and DS genotypes under drought and control conditions. The identified DATs and DAPs were found to participate in various metabolic pathways and biological processes. The up-regulated genes and proteins were involved mainly in glycolysis/gluconeogenesis and galactose metabolism in DT genotype under drought stress. On contrary, the DS genotype exhibited down-regulation of glycolysis/gluconeogenesis associated genes and proteins under drought stress. In addition, metabolites involved in carbohydrate metabolism were found to be differentially abundant in both DT and DS genotypes. The carbohydrate metabolism is a major pathway regulating cellular carbon and energy requirement for plant survival under drought stress, and genes and proteins encoding the key enzymes of carbohydrate metabolism were found to be differentially expressed under drought stress in chickpea (Khanna et al., 2014). Indeed, sugars are considered an important player in mitigating abiotic stress tolerance in plants (Keunen et al., 2013; Gangola and Ramadoss, 2018). These results demonstrated the important role of carbohydrate metabolism to withstand drought stress in the DT genotype. The amino acids are the precursors of secondary metabolites and signaling molecules, and promote ATP production and detoxification under abiotic stress (Batista-Silva et al., 2019). In our study, up-regulation of several genes/proteins of amino acid metabolism, and differential abundance of amino acids in DT and DS genotypes was suggested to be involved in drought response. Higher amino acid accumulation is a general attribute to drought stress in the plants (Martinelli et al., 2007). Furthermore, hormone signaling and its cross-talk improve abiotic stress tolerance by inducing stress-responsive genes (Ku et al., 2018). Therefore, up-regulation of hormone signaling related genes and proteins in DT(C)/DS(C) might contribute to encountering drought stress. Overall, the activation of stress-responsive genes, proteins and metabolites might be involved in the better adaptation of DT genotype to drought stress.

### 4.2 TFs confer genotype-specific response under drought stress

TFs are important regulators contributing to abiotic stress tolerance in the plants (Yoon et al., 2020). In our study, up-regulation of members of GNAT, GRAS, C3H, HB, bZIP, PLATZ, sigma70-like, CCAAT, WRKY, Aux/IAA and C2H2-Dof TF families in DT(C)/DS(C) suggested the genotype-specific involvement of TFs. A significant increase in the expression of GNAT family members, histone acetyltransferase (HAT) and acetylation of histone H3 and H4 has been reported in rice under drought stress (Fang et al., 2014). Importantly, drought-induced genome-wide histone acetylation changes the expression of drought-responsive genes (Li et al., 2021). The over-expression of *OsGRAS23* and *TaWRKY2* induced drought tolerance in rice and wheat, respectively (Xu et al., 2015; Gao et al., 2018). Likewise, over-expression of homeobox (HB) genes, *OsHOX24* and *OsWOX13* enhanced abiotic stress tolerance in rice (Bhattacharjee et al., 2016; Minh-Thu et al., 2018). Rice *OsbZIP62*, involved in ABA signaling, positively regulates the expression of drought-responsive genes (Yang et al., 2019). However, over-expression and RNAi knockdown of *OsbZIP71* generated drought-tolerant and sensitive phenotype, respectively (Liu et al., 2014). The C3H ZF-TF (*AetTZF1*) has been suggested to enhance drought tolerance by promoting root growth in *Arabidopsis* (Jiang et al., 2014), and C2H2-ZFPs provided abiotic and oxidative stress tolerance (Huang et al., 2009). Likewise, WRKY TF provided adaptation to abiotic stress tolerance by modulating stress-responsive genes (Sen et al., 2017). Therefore, the differential abundance of TFs can contribute to better transcriptional control in multiple regulatory pathways for enhanced drought tolerance in DT genotype.

### 4.3 Implication of *QTL-hotspot* region associated factors in drought tolerance

*QTL-hotspot* region plays a major role in drought tolerance in chickpea, and its introgression in an elite variety (JG 11) improved the root traits and drought tolerance (Varshney et al., 2013b). Integration of *QTL* information and co-expression network analysis has been exploited successfully for the identification of potential candidate genes associated with the maize kernel starch content (Lin et al., 2019a). We applied a similar strategy for the identification of drought-responsive candidate genes and proteins within the *QTL-hotspot*. The *QTL-hotspot* region associated genes and proteins in DT(C)/DS(C) have been investigated for their involvement in drought tolerance/response in previous studies. Among the identified genes of co-expression *QTL-hotspot*, cytochrome P450 (CYP), *OsDSS1* is known to be involved in growth and drought stress response in rice (Tamiru et al., 2015). Drought-responsive miR-114 and its target gene, namely stabilizer of iron transporter SufD/polynucleotidyl transferase, participated in drought tolerance in *Sorghum bicolor* (Katiyar et al., 2015). Likewise, *HORVU2Hr1G023890* encoding myosin-J heavy chain is associated with drought tolerance during seed germination in barley (Thabet et al., 2018). The over-expression of receptor-like kinases (RLKs) enhanced abiotic stress tolerance (Osakabe et al., 2010), and cysteine-rich receptor-like protein kinase 5 (CRK5) improved drought tolerance in *Arabidopsis* (Lu et al., 2016). Furthermore, over-expression of copper amine oxidase 1 mediates proper xylem development and differentiation in *Arabidopsis* roots, and xylem morphology determines drought response (Ghuge et al., 2015). Moreover, the co-expression *QTL-hotspot* region associated up-regulated TF, DREB (*Ca_04504*) is known to contribute in drought tolerance (Haake et al., 2002; Zhang et al., 2009). Similarly, up-regulated *pQTL*-*hotspot* associated protein, oligo-saccharyltransferase complex/magnesium transporter family protein (Ca_04632), has been reported previously to participate in drought tolerance (Farid et al., 2013). These results suggested that the *QTL*-*hotspot* region associated drought-responsive genes, proteins and TFs contribute to improved drought tolerance in the DT genotype.

### 4.4 Lipid signaling, glutathione metabolism and glycolysis/gluconeogenesis pathways are involved in drought tolerance

PI signaling is crucial for regulating cellular homeostasis during abiotic stress (Hou et al., 2016). Under stress conditions, the PI signaling pathway influences membrane integrity and cellular signaling *via* phosphorylation and de-phosphorylation of its related enzymes by kinases and phosphatases, respectively (Heilmann, 2016). The co-expression of various kinases and phosphatases encoding genes and proteins suggested a more pronounced regulation of PI signaling in DT genotype under drought. It has been demonstrated that the identified kinases and phosphatases are involved in cell cycle regulation, histone modifications, cytoskeleton organization and protein sorting in response to abiotic stress (Williams et al., 2005; Pribat et al., 2011; Dieck et al., 2012; Akhter et al., 2016). Further, *FAB1D/PI3P5K* allows protein trafficking in cortical and stele cells for proper root development (Hirano and Munnik, 2015), and *OsDGK1* restores lateral root (LR) density and seminal root (SR) formation (Yuan et al., 2019). Accordingly, we also suggest the involvement of kinases and phosphatases in better root development and hence drought tolerance in the DT genotype. The myo-inositol acts as an osmoprotectant and maintains plant adaptation to drought stress (Li et al., 2020). The over-expression of IMPase increases myo-inositol content and results in an enhanced abiotic stress tolerance (Zhang et al., 2017). Likewise, up-regulation of IMPase (Ca_05134) leading to accumulation of myo-inositol in DT(D/C) suggested its specific role in drought stress tolerance.

Glutathione metabolism is known to be involved in maintaining cellular redox homeostasis during drought stress (Nahar et al., 2015). This adaptive mechanism encounters oxidative damage by utilizing various ROS scavengers (antioxidants) and redox responsive genes (Aquilano et al., 2014). The glutathione metabolism related genes and proteins are well known for regulating cellular redox homeostasis during abiotic stress (Chen et al., 2003). We observed differential regulation of glutathione metabolism in both the genotypes, and genes and proteins of glutathione metabolism were found to be co-expressed specifically in DT(D). The identified genes have previously been reported to contribute to abiotic stress tolerance. For example, the over-expression of glutathione metabolism related gene/proteins, including *GST*, *G6PDH*, *PGDH*, *APX*, *GSTU* and *GPX* improved drought tolerance in the transgenic plants (Badawi et al., 2004; Miao et al., 2006; Ji et al., 2010; Lin et al., 2013; Srivastava et al., 2019). The peptidases and GCL positively regulate plant tolerance to abiotic stress *via* regulating cellular redox homeostasis (Hicks et al., 2007; Simova-Stoilova et al., 2009). Likewise, GPx/Prx system efficiency regulates H_2_O_2_ concentration in balancing the cellular glutathione level (Molavian et al., 2015). Moreover, PGDH and G6PDH maintain cellular redox homeostasis by elevating NADPH/NADP^+^ levels in response to drought and salt stresses (Chen et al., 2003). In accordance, co-expression of glutathione metabolism related gene and proteins in DT(D) might explain, in large extent, the enhanced drought tolerance in the DT genotype. In addition, differential abundance of GST (*Ca_12001*), GSTU (*Ca_05354*) and G6PDH (Ca_10123) may further contribute to improved drought tolerance in DT. Higher amino acids level has been suggested to improve drought performance (Hildebrandt, 2018), and regulating meristem activity and root architecture during drought stress (Walch-Liu et al., 2006). Similarly, amino acids accumulation *via* glutathione metabolism is indicative of stress-adaptive responses in DT. Considering the intricate regulation *via* stress-responsive genes, proteins and metabolites, an improved glutathione metabolic pathway seems to contribute to the enhanced drought tolerance in the DT genotype.

The glycolysis/gluconeogenesis is an important metabolic pathway that regulates carbohydrate metabolism under drought stress (Zhu et al., 2019). Glycolysis/gluconeogenesis associated co-expressed genes and proteins identified in DT(D) are known to be involved in abiotic stress tolerance (Pan et al., 2016; Lin et al., 2019b; Zeng et al., 2019). Among the identified co-expressed genes and proteins in DT(D), *PK*, *PGAM*, *LDH*, *PFK*, *TPI* and *GPI* have been reported to express differentially under drought (Sharma et al., 2012; Khanna et al., 2014; Pan et al., 2016; Yao and Wu, 2016; Li et al., 2019a). Moreover, *ENO* is known to promote transcription of glycolysis related genes under abiotic stress (Lee et al., 2002). Likewise, *GALM* maintains drought adaptation and recovery (D’Andrea et al., 2015), and FBPase undergoes post-translation modification to regulate glycolysis during drought stress (Harn and Daie, 1992). Moreover, over-expression of *OsPgk2a*-P, *ScALDH21, ZmPCK2*, *LDH* and *TraeALDH7B1-5A* confer improved abiotic stress tolerance (Chen et al., 2015; Joshi et al., 2016; Yang et al., 2016; Jain et al., 2020; Jiang et al., 2022). Notably, the role of co-expressed genes in DT(D), namely *Dlat* and AAE, have not been reported in drought response/tolerance in plants yet. Therefore, further studies are required to elucidate their role in drought response. The up-regulation of FBPase (*Ca_00102*), PEPCK1 (*Ca_11524*), GALM-like (Ca_15348) and PGAM (Ca_09253) in DT(D/C) may further contribute to enhanced drought tolerance in the DT genotype. Glucose is a precursor of glycolysis/gluconeogenesis pathway and, is necessary for coordinate regulation of both glycolysis and gluconeogenesis. Moreover, D-glucose is known to induce the expression of abscisic acid (ABA) signaling related genes (Dekkers et al., 2008; Fukumoto et al., 2013), and certainly ABA is critical for controlling abiotic stress responses (Tuteja, 2007). However, D-mannitol is an important osmolyte and compatible solutes providing enhanced tolerance to salt and drought stress (Pujni et al., 2007). Accordingly, accumulation and differential abundance of D-mannitol and D-glucose in DT genotype is an indicative of improved drought tolerance. These results highlighted that DT genotype possesses an improved carbohydrate metabolism to withstand drought stress. Altogether, the synergistic response of co-expressed genes, proteins and metabolites involved in PI signaling pathway, glutathione metabolism and carbohydrate metabolism circumvent the enhanced drought stress tolerance in the DT genotype.

## 5 Conclusions

To provide a better understanding of drought tolerance, we performed integrated multi-omics analysis in two chickpea genotypes with contrasting responses to drought stress. The WGCNA analysis revealed the co-expressed genes, proteins and metabolites in DT and DS genotypes under control and/or drought conditions. The integrated transcriptome, proteome, and metabolome analysis identified important stress-responsive pathways, including PI signaling, glutathione metabolism and glycolysis/gluconeogenesis that plausibly, in large part, contribute to the drought tolerance in chickpea. Moreover, up-regulated genes, proteins and TFs associated with the *QTL*-*hotspot* region seem to determine drought tolerance in the DT genotype. Overall, these results provide new insights into drought stress response in chickpea and reveal candidate genes/proteins that can serve as potential targets for further functional characterization and improving chickpea drought tolerance *via* genome engineering approaches.

## Data availability statement

The datasets presented in this study can be found in online repositories. The transcriptome data generated in this study have been submitted to the GEO at NCBI under the series accession number GSE193077. Proteome data have been submitted to PRIDE database under the project accession number PXD041434 and metabolome data have been submitted to MetaboLights database with study identifier number MTBLS7641.

## Author contributions

MJ and RG conceived, designed, and supervised the study. RG performed stress experiments, collected the tissue materials and performed RNA sequencing. VS performed proteome and metabolome experiments, data analyses, and integrated data analyses. KG performed transcriptome data analysis. SS performed RT-qPCR analysis. VS, MJ, and RG wrote the manuscript with inputs from others. All authors read and approved the final version manuscript. All authors contributed to the article.

## References

[B1] Akhter S. Uddin M. N. Jeong I. S. Kim D. W. Liu X. M. Bahk J. D. (2016). Role of arabidopsis Atpi4kγ3, a type II phosphoinositide 4-kinase, in abiotic stress responses and floral transition. Plant Biotechnol. J. 14, 215–230. doi: 10.1111/Pbi.12376 25879253 PMC11389056

[B2] Amiour N. Imbaud S. Clément G. Agier N. Zivy M. Valot B.. (2012). The use of metabolomics integrated with transcriptomic and proteomic studies for identifying key steps involved in the control of nitrogen metabolism in crops such as maize. J. Exp. Bot. 63, 5017–5033. doi: 10.1093/Jxb/Ers186 22936829

[B3] Aquilano K. Baldelli S. Ciriolo M. (2014). Glutathione: new roles in redox signaling for an old antioxidant. Front. Pharma. 5. doi: 10.3389/Fphar.2014.00196 PMC4144092 25206336

[B4] Badawi G. H. Kawano N. Yamauchi Y. Shimada E. Sasaki R. Kubo A.. (2004). Over-expression of ascorbate peroxidase in tobacco chloroplasts enhances the tolerance to salt stress and water deficit. Physiol. Plant 121, 231–238. doi: 10.1111/J.0031-9317.2004.00308.X 15153190

[B5] Banerjee A. Roychoudhury A. (2019). “Role of glutathione in plant abiotic stress tolerance,” in Reactive oxygen, nitrogen and sulfur species in plants. Eds. Hasanuzzaman E. M. Fotopoulos V. Nahar K. Fujita M. (USA: John Wiley & Sons, Ltd), p 159–p 172.

[B6] Barua P. Lande N. V. Subba P. Gayen D. Pinto S. Keshava Prasad T. S.. (2019). Dehydration-responsive nuclear proteome landscape of chickpea *(Cicer Arietinum L.*) reveals phosphorylation-mediated regulation of stress response. Plant Cell Env. 42, 230–244. doi: 10.1111/Pce.13334 29749054

[B7] Batista-Silva W. Heinemann B. Rugen N. Nunes-Nesi A. Araújo W. L. Braun H.-P.. (2019). The role of amino acid metabolism during abiotic stress release. Plant Cell Env. 42, 1630–1644. doi: 10.1111/Pce.13518 30632176

[B8] Bhaskarla V. Zinta G. Ford R. Jain M. Varshney R. K. Mantri N. (2020). Comparative root transcriptomics provide insights into drought adaptation strategies in chickpea *(Cicer Arietinum L.*). Int. J. Mol. Sci. 21, 1781. doi: 10.3390/Ijms21051781 32150870 PMC7084756

[B9] Bhattacharjee A. Khurana J. P. Jain M. (2016). Characterization of rice homeobox genes, Oshox22 and Oshox24, and over-expression of Oshox24 in transgenic arabidopsis suggest their role in abiotic stress response. Front. Plant Sci. 7. doi: 10.3389/Fpls.2016.00627 PMC4862318 27242831

[B10] Bittencourt C. B. da Silva T. L. C. Neto J. C. R. Vieira L. R. Leão A. P. Ribeiro J. A. A.. (2022). Insights from a multi-omics integration (MOI) study in oil palm *(Elaeis guineensis* jacq.) response to abiotic stresses: part one-salinity. Plants 11 (13), 1755. doi: 10.3390/plants11131755 35807707 PMC9269341

[B11] Cevik S. Akpinar G. Yildizli A. Kasap M. Karaosmanoglu K. Unyayar S. (2019). Comparative physiological and leaf proteome analysis between drought-tolerant chickpea *Cicer Reticulatum* and drought-sensitive chickpea *C. Arietinum* . J. Biosci. 44 (1), 20. doi: 10.1007/S12038-018-9836-4 30837371

[B12] Chen K. M. Gong H. J. Chen G. C. Wang S. M. Zhang C. L. (2003). Up-regulation of glutathione metabolism and changes in redox status involved in adaptation of reed (*Phragmites Communis*) ecotypes to drought-prone and saline habitats. J. Plant Physiol. 160, 293–301. doi: 10.1078/0176-1617-00927 12749086

[B13] Chen J. Wei B. Li G. Fan R. Zhong Y. Wang X. (2015). *Traealdh7b1-5A* , encoding aldehyde dehydrogenase 7 in wheat, confers improved drought tolerance in arabidopsis. Planta 242, 137–151. doi: 10.1007/S00425-015-2290-8 25893867

[B14] Chin E. L. Ramsey J. S. Mishchuk D. O. Saha S. Foster E. Chavez J. D.. (2020). Longitudinal transcriptomic, proteomic, and metabolomic analyses of *Citrus sinensis* (L.) osbeck graft-inoculated with *Candidatus* liberibacter asiaticus. J. Proteome Res. 19 (2), 719–732. doi: 10.1021/acs.jproteome.9b00616 31885275

[B15] Cox J. Neuhauser N. Michalski A. Scheltema R. A. Olsen J. V. Mann M. (2011). Andromeda: a peptide search engine integrated into the maxquant environment. J. Proteome Res. 10, 1794–1805. doi: 10.1021/Pr101065j 21254760

[B16] D’Andrea R. M. Triassi A. Casas M. I. Andreo C. S. Lara M. V. (2015). Identification of genes involved in the drought adaptation and recovery in portulaca oleracea by differential display. Plant Physiol. Biochem. 90, 38–49. doi: 10.1016/J.Plaphy.2015.02.023 25767913

[B17] Dekkers B. J. W. Schuurmans J. A. M. J. Smeekens S. C. M. (2008). Interaction between sugar and abscisic acid signalling during early seedling development in arabidopsis. Plant Mol. Biol. 67, 151–167. doi: 10.1007/S11103-008-9308-6 18278579 PMC2295253

[B18] Dieck C. B. Wood A. Brglez I. Rojas-Pierce M. Boss W. F. (2012). Increasing phosphatidylinositol (4,5) bisphosphate biosynthesis affects plant nuclear lipids and nuclear functions. Plant Physiol. Biochem. 57, 32–44. doi: 10.1016/J.Plaphy.2012.05.011 22677448 PMC3601448

[B19] Fang H. Liu X. Thorn G. Duan J. Tian L. (2014). Expression analysis of histone acetyltransferases in rice under drought stress. Biochem. Biophys. Res. Commun. 443, 400–405. doi: 10.1016/J.Bbrc.2013.11.102 24309107

[B20] FAO (2016). Statistical database (Rome, Italy: Food And Agriculture Organization Of The United Nations). Available at: Http//Www.Apps.Fao.Org.

[B21] Farid A. Malinovsky F. G. Veit C. Schoberer J. Zipfel C. Strasser R. (2013). Specialized roles of the conserved subunit OST3/6 of the oligosaccharyltransferase complex in innate immunity and tolerance to abiotic stresses. Plant Physiol. 162, 24–38. doi: 10.1104/Pp.113.215509 23493405 PMC3641206

[B22] Fukumoto T. Kano A. Ohtani K. Inoue M. Yoshihara A. Izumori K.. (2013). Phosphorylation of d-allose by hexokinase involved in regulation of Osabf1 expression for growth inhibition in *Oryza Sativa* l. Planta 237, 1379–1391. doi: 10.1007/S00425-013-1853-9 23397192

[B23] Gangola M. P. Ramadoss B. R. (2018). “Sugars play a critical role in abiotic stress tolerance in plants,” in Biochemical, physiological and molecular avenues for combating abiotic stress tolerance in plants. Ed. Wani S. H. (Academic Press), p 17–p 38.

[B24] Gao H. Wang Y. Xu P. Zhang Z. (2018). Overexpression of a WRKY transcription factor Tawrky2 enhances drought stress tolerance in transgenic wheat. Front. Plant Sci. 9. doi: 10.3389/Fpls.2018.00997 PMC6090177 30131813

[B25] Garg R. Bhattacharjee A. Jain M. (2015). Genome-scale transcriptomic insights into molecular aspects of abiotic stress responses in chickpea. Plant Mol. Biol. Rep. 33, 388–400. doi: 10.1007/S11105-014-0753-X

[B26] Garg R. Sahoo A. Tyagi A. K. Jain M. (2010). Validation of internal control genes for quantitative gene expression studies in chickpea (*Cicer Arietinum L.*). Biochem. Biophy. Res. Commun. 396, 283–288. doi: 10.1016/J.Bbrc.2010.04.079 20399753

[B27] Garg R. Shankar R. Thakkar B. Kudapa H. Krishnamurthy L. Mantri N.. (2016). Transcriptome analyses reveal genotype- and developmental stage-specific molecular responses to drought and salinity stresses in chickpea. Sci. Rep. 6, 19228. doi: 10.1038/Srep19228 26759178 PMC4725360

[B28] Gaur P. M. Krishnamurthy L. Kashiwagi J. (2008). Improving drought-avoidance root traits in chickpea (Cicer arietinum l.) -current status of research At ICRISAT. Plant Pro. Sci. 11, 3–11. doi: 10.1626/Pps.11.3

[B29] Ghuge S. A. Carucci A. Rodrigues-Pousada R. A. Tisi A. Franchi S. Tavladoraki P.. (2015). The apoplastic copper amine Oxidase1 mediates jasmonic acid-induced protoxylem differentiation in arabidopsis roots. Plant Physiol. 168, 690–707. doi: 10.1104/Pp.15.00121 25883242 PMC4453780

[B30] Gupta S. Mishra S. K. Misra S. Pandey V. Agrawal L. Nautiyal C. S.. (2020). Revealing the complexity of protein abundance in chickpea root under drought-stress using a comparative proteomics approach. Plant Physiol. Biochem. 151, 88–102. doi: 10.1016/J.Plaphy.2020.03.005 32203884

[B31] Haake V. Cook D. Riechmann J. L. Pineda O. Thomashow M. F. Zhang J. Z. (2002). Transcription factor CBF4 is a regulator of drought adaptation in arabidopsis. Plant Physiol. 130, 639–648. doi: 10.1104/Pp.006478 12376631 PMC166593

[B32] Harn C. Daie J. (1992). Regulation of the cytosolic fructose-1,6-Bisphosphatase by post-translational modification and protein level in drought-stressed leaves of sugarbeet. Plant Cell Physiol. 33, 763–770. doi: 10.1093/Oxfordjournals.Pcp.A078316

[B33] Heilmann I. (2016). Phosphoinositide signaling in plant development. Dev. 143, 2044–2055. doi: 10.1242/Dev.136432 27302395

[B34] Hicks L. M. Cahoon R. E. Bonner E. R. Rivard R. S. Sheffield J. Jez J. M. (2007). Thiol-based regulation of redox-active glutamate-cysteine ligase from *Arabidopsis Thaliana* . Plant Cell 19, 2653–2661. doi: 10.1105/Tpc.107.052597 17766407 PMC2002632

[B35] Hildebrandt T. M. (2018). Synthesis versus degradation: directions of amino acid metabolism during arabidopsis abiotic stress response. Plant Mol. Biol. 98, 121–135. doi: 10.1007/S11103-018-0767-0 30143990

[B36] Hirano T. Munnik T. (2015). Phosphatidylinositol 3-phosphate 5-kinase, FAB1/Pikfyve kinase mediates endosome maturation to establish endosome-cortical microtubule interaction in arabidopsis. Plant Physiol. 169, 1961–1974. doi: 10.1104/Pp.15.01368 26353760 PMC4634102

[B37] Hou Q. Ufer G. Bartels D. (2016). Lipid signalling in plant responses to abiotic stress. Plant Cell Env. 39, 1029–1048. doi: 10.1111/Pce.12666 26510494

[B38] Huang J. Sun S. J. Xu D. Q. Yang X. Bao Y. M. Wang Z. F.. (2009). Increased tolerance of rice to cold, drought and oxidative stresses mediated by the overexpression of a gene that encodes the zinc finger protein ZFP245. Biochem. Biophy. Res. Comm 389, 556–561. doi: 10.1016/J.Bbrc.2009.09.032 19751706

[B39] Jaganathan D. Thudi M. Kale S. Azam S. Roorkiwal M. Gaur P. M.. (2015). Genotyping-By-Sequencing based intra-specific genetic map refines a ‘‘QTL-hotspot” region for drought tolerance in chickpea. Mol. Gen. Genom 290, 559–571. doi: 10.1007/S00438-014-0932-3 PMC4361754 25344290

[B40] Jain M. Aggarwal S. Nagar P. Tiwari R. Mustafiz A. (2020). A d-lactate dehydrogenase from rice is involved in conferring tolerance to multiple abiotic stresses by maintaining cellular homeostasis. Sci. Rep. 10, 12835. doi: 10.1038/S41598-020-69742-0 32732944 PMC7393112

[B41] Ji W. Zhu Y. Li Y. Yang L. Zhao X. Cai H.. (2010). Over-expression of a glutathione s-transferase gene, *Gsgst* , from wild soybean (*Glycine Soja*) enhances drought and salt tolerance in transgenic tobacco. Biotechnol. Lett. 32, 1173–1179. doi: 10.1007/S10529-010-0269-X 20383560

[B42] Jiang A. L. Xu Z. S. Zhao G. Y. Cui X. Y. Chen M. Li L. C.. (2014). Genome-wide analysis of the C3H zinc finger transcription factor family and drought responses of members in aegilops tauschii. Plant Mol. Biol. Rep. 32, 1241–1256. doi: 10.1007/S11105-014-0719-Z

[B43] Jiang D. Zhang H. Cai H. Gao Z. Chen G. (2022). Overexpression of Zmpck2, a phosphoenolpyruvate carboxykinase gene from maize confers enhanced tolerance to water deficit stress in rice. Plant Sci. 317, 111195. doi: 10.1016/J.Plantsci.2022.111195 35193744

[B44] Joshi R. Karan R. Singla-Pareek S. Pareek A. (2016). Ectopic expression of pokkali phosphoglycerate kinase-2 ( *Ospgk2-P* ) improves yield in tobacco plants under salinity stress. Plant Cell Rep. 35, 27–41. doi: 10.1007/S00299-015-1864-Z 26408146

[B45] Jukanti A. K. Gaur P. M. Gowda C. L. L. Chibbar R. N. (2012). Nutritional quality and health benefits of chickpea *(Cicer Arietinum L.* ). Brit. J. Nutri. 108, S11–S26. doi: 10.1017/S0007114512000797 22916806

[B46] Kashiwagi J. Krishnamurthy L. Gaur P. Upadhyaya H. Varshney R. Tobita S. (2013). Traits of relevance to improve yield under terminal drought stress in chickpea *(C. Arietinum* l.). Field Crops Res. 145, 88–95. doi: 10.1016/J.Fcr.2013.02.011

[B47] Kashiwagi J. Krishnamurthy L. Purushothaman R. Upadhyaya H. D. Gaur P. M. Gowda C. L. L.. (2015). Scope for improvement of yield under drought through the root traits in chickpea *(Cicer Arietinum L.* ). Field Crops Res. 170, 47–54. doi: 10.1016/J.Fcr.2014.10.003

[B48] Kashiwagi J. Krishnamurthy L. Upadhyaya H. D. Krishna H. Chandra S. Vadez V.. (2005). Genetic variability of drought-avoidance root traits in the mini-core germplasm collection of chickpea *(Cicer Arietinum* l. ). Euphy 146, 213–222. doi: 10.1007/S10681-005-9007-1

[B49] Katiyar A. Smita S. Muthusamy S. K. Chinnusamy V. Pandey D. M. Bansal K. C. (2015). Identification of novel drought-responsive micrornas and trans-acting sirnas from *Sorghum Bicolor* (L.) moench by high-throughput sequencing analysis. Front. Plant Sci. 6. doi: 10.3389/Fpls.2015.00506 PMC4504434 26236318

[B50] Kausar R. Wang X. Komatsu S. (2022). Crop proteomics under abiotic stress: from data to insights. Plants 11 (21), 2877. doi: 10.3390/plants11212877 36365330 PMC9657731

[B51] Kelly R. S. Chawes B. L. Blighe K. Virkud Y. V. Croteau-Chonka D. C. McGeachie M. J. (2018). An integrative transcriptomic and metabolomic study of lung function in children with asthma. Chest. 154 (2), 335–348. doi: 10.1016/j.chest.2018.05.038 29908154 PMC6689076

[B52] Keunen E. Peshev D. Vangronsheld J. Van Den Ende W. Cuypers A. (2013). Plant sugars are crucial players in the oxidative challenge during abiotic stress: extending the traditional concept. Plant Cell Env. 36, 1242–1255. doi: 10.1111/Pce.12061 23305614

[B53] Khan N. Bano A. Rahman M. A. Rathinasabapathi B. Babar M. A. (2019). UPLC-HRMS-Based untargeted metabolic profiling reveals changes in chickpea *(Cicer Arietinum*) metabolome following long-term drought stress. Plant Cell Env. 42, 115–132. doi: 10.1111/Pce.13195 29532945 PMC7379973

[B54] Khanna S. Taxak P. Jain P. Saini R. Srinivasan R. (2014). Glycolytic enzyme activities and gene expression in cicer arietinum exposed to water-deficit stress. App. Biochem. Biotechnol. 173, 2241–2253. doi: 10.1007/S12010-014-1028-6 25008554

[B55] Kosova K. Vítamvas P. Prášil I. T. Klíma M. Renaut J. (2021). Plant proteoforms under environmental stress: functional proteins arising from a single gene. Front. Plant Sci. 12 (793113). doi: 10.3389/fpls.2021.793113 PMC8712444 34970290

[B56] Kosova K. Vítamvas P. Urban M. O. Prášil I. T. Renaut J. (2018). Plant abiotic stress proteomics: the major factors determining alterations in cellular proteome. Front. Plant Sci. 9 (122). doi: 10.3389/fpls.2018.00122 PMC5810178 29472941

[B57] Krishnamurthy L. Kashiwagi J. Gaur P. Upadhyaya H. Vadez V. (2010). Sources of tolerance to terminal drought in the chickpea *(Cicer Arietinum* l.) minicore germplasm. Field Crops Res. 119, 322–330. doi: 10.1016/J.Fcr.2010.08.002

[B58] Ku Y. Sintaha M. Cheung M. Lam H. (2018). Plant hormone signaling crosstalks between biotic and abiotic stress responses. Int. J. Mol. Sci. 19 (10), 3206. doi: 10.3390/Ijms19103206 30336563 PMC6214094

[B59] Kudapa H. Garg V. Chitikineni A. Varshney R. K. (2018). The RNA-Seq-Based high resolution gene expression atlas of chickpea *(Cicer Arietinum L.* ) reveals dynamic spatio-temporal changes associated with growth and development. Plant Cell Env. 41, 2209–2225. doi: 10.1111/Pce.13210 29637575

[B60] Kumar M. Chauhan A. S. Kumar M. Yusuf M. A. Sanyal I. Chauhan P. S. (2019). Transcriptome sequencing of chickpea (Cicer arietinum l.) genotypes for identification of drought-responsive genes under drought stress condition. Plant Mol. Biol. Rep. 37, 186–203. doi: 10.1007/S11105-019-01147-4

[B61] Langfelder P. Horvath S. (2008). WGCNA: an r package for weighted correlation network analysis. BMC Bioinfo. 9 (559). doi: 10.1186/1471-2105-9-559 PMC2631488 19114008

[B62] Leão A. P. Bittencourt C. B. da Silva T. L. C. Neto J. C. R. Braga Í.O. Vieira L. R.. (2022). Insights from a multi-omics integration (MOI) study in oil palm *(Elaeis guineensis* jacq.) response to abiotic stresses: part two-drought. Plants 11 (20), 2786. doi: 10.3390/plants11202786 36297811 PMC9611107

[B63] Lee H. Guo Y. Ohta M. Xiong L. Stevenson B. Zhu J. (2002). LOS2, a genetic locus required for cold-responsive gene transcription encodes a bi-functional enolase. EMBO J. 21, 2692–2702. doi: 10.1093/Emboj/21.11.2692 12032082 PMC126021

[B64] Li Z. Fu J. Shi D. Peng Y. (2020). Myo-inositol enhances drought tolerance in creeping bentgrass through alteration of osmotic adjustment, photosynthesis, and antioxidant defense. Crop Sci. 60, 2149–2158. doi: 10.1002/Csc2.20186

[B65] Li P. Yang H. Wang L. Liu H. Huo H. Zhang C.. (2019a). Physiological and transcriptome analyses reveal short-term responses and formation of memory under drought stress in rice. Front. Gent 10. doi: 10.3389/Fgene.2019.00055 PMC6375884 30800142

[B66] Li S. He X. Gao Y. Zhou C. Chiang V. L. Li W. (2021). Histone acetylation changes in plant response to drought stress. Genes 12, 1409. doi: 10.3390/Genes12091409 34573391 PMC8468061

[B67] Li T. Yun Z. Wu Q. Qu H. Duan X. Jiang Y. (2019b). Combination of transcriptomic, proteomic, and metabolomic analysis reveals the ripening mechanism of banana pulp. Biomolecules. 9 (10), 523. doi: 10.3390/biom9100523 31548496 PMC6843284

[B68] Lin Y. Li W. Zhang Y. Xia C. Liu Y. Wang C.. (2019b). Identification of Genes/Proteins related to submergence tolerance by transcriptome and proteome analyses in soybean. Sci. Rep. 9, 14688. doi: 10.1038/S41598-019-50757-1 31604973 PMC6789146

[B69] Lin Y. Lin S. Guo H. Zhang Z. Chen X. (2013). Functional analysis of *Psg6pdh* , a cytosolic glucose-6-Phosphate dehydrogenase gene from *Populus Suaveolens* , and its contribution to cold tolerance improvement in tobacco plants. Biotechnol. Lett. 35, 1509–1518. doi: 10.1007/S10529-013-1226-2 23690038

[B70] Lin F. Zhou L. He B. Zhang X. Dai H. Qian Y.. (2019a). QTL mapping for maize starch content and candidate gene prediction combined with Co-expression network analysis. Theo. Appl. Gent. 132, 1931–1941. doi: 10.1007/S00122-019-03326-Z 30887095

[B71] Liu P. Luo J. Zheng Q. Chen Q. Zhai N. Xu S.. (2020). Integrating transcriptome and metabolome reveals molecular networks involved in genetic and environmental variation in tobacco. DNA Res. 27 (2), 1–16. doi: 10.1093/dnares/dsaa006 PMC7320822 32324848

[B72] Liu C. Mao B. Ou S. Wang W. Liu L. Wu Y.. (2014). *Osbzip71* , a bzip transcription factor, confers salinity and drought tolerance in rice. Plant Mol. Biol. 84, 19–36. doi: 10.1007/S11103-013-0115-3 23918260

[B73] Lu K. Liang S. Wu Z. Bi C. Yu Y. T. Wang X. F.. (2016). Overexpression of an arabidopsis cysteine-rich receptor-like protein kinase, *CRK5* , enhances abscisic acid sensitivity and confers drought tolerance. J. Exp. Bot. 67, 5009–5027. doi: 10.1093/Jxb/Erw266 27406784 PMC5014153

[B74] Martinelli T. Whittaker A. Bochicchio A. Vazzana C. Suzuki A. Masclaux-Daubresse C. (2007). Amino acid pattern and glutamate metabolism during dehydration stress in the ‘Resurrection’ plant sporobolus stapfianus: a comparison between desiccation-sensitive and desiccation-tolerant leaves. J. Exp. Bot. 58, 3037–3046. doi: 10.1093/Jxb/Erm161 17901195

[B75] Mashaki M. Garg V. Nasrollahnezhad Ghomi A. A. Kudapa H. Chitikineni A. Zaynali Nezhad K.. (2018). RNA-Seq analysis revealed genes associated with drought stress response in kabuli chickpea *(Cicer Arietinum *L.). PloS One 13, E0199774. doi: 10.1371/Journal.Pone.0199774 29953498 PMC6023194

[B76] Miao Y. Lv D. Wang P. Wang X. C. Chen J. Miao C.. (2006). An arabidopsis glutathione peroxidase functions as both a redox transducer and a scavenger in abscisic acid and drought stress responses. Plant Cell 18, 2749–2766. doi: 10.1105/Tpc.106.044230 16998070 PMC1626619

[B77] Minh-Thu P. T. Kim J. S. Chae S. Jun K. M. Lee G. S. Kim D. E.. (2018). A WUSCHEL homeobox transcription factor, Oswox13, enhances drought tolerance and triggers early flowering in rice. Mol. Cells 41, 781–798. doi: 10.14348/Molcells.2018.0203 30078233 PMC6125423

[B78] Molavian H. Madani Tonekaboni A. Kohandel M. Sivaloganathan S. (2015). The synergetic coupling among the cellular antioxidants glutathione Peroxidase/Peroxiredoxin and other antioxidants and its effect on the concentration of H2O2. Sci. Rep. 5, 13620. doi: 10.1038/Srep13620

[B79] Moons A. (2005). Regulatory and functional interactions of plant growth regulators and plant glutathione s-transferases (Gsts). Vita. Hormones. 72, 155–202. doi: 10.1016/S0083-6729(05)72005-7 16492471

[B80] Moreno J. C. Martinez-Jaime S. Kosmacz M. Sokolowska E. M. Schulz P. Fischer A.. (2021). A multi-OMICs approach sheds light on the higher yield phenotype and enhanced abiotic stress tolerance in tobacco lines expressing the carrot *lycopene* β *-cyclase1* gene. Front. Plant Sci. 12. doi: 10.3389/fpls.2021.624365 PMC7893089 33613605

[B81] Nahar K. Hasanuzzaman M. Alam M. M. Fujita M. (2015). Glutathione-induced drought stress tolerance in mung bean: coordinated roles of the antioxidant defence and methylglyoxal detoxification systems. AOB Plants 7, Plv069. doi: 10.1093/Aobpla/Plv069 26134121 PMC4526754

[B82] Nawae W. Shearman J. R. Tangphatsornruang S. Punpee P. Yoocha T. Sangsrakru D.. (2020). Differential expression between drought-tolerant and drought-sensitive sugarcane under mild and moderate water stress as revealed by a comparative analysis of leaf transcriptome. Peer J. 8, E9608. doi: 10.7717/Peerj.9608 33240580 PMC7676377

[B83] Nayyar H. Kaur S. Singh S. Upadhyaya H. D. (2006). Differential sensitivity of desi (Small-seeded) and kabuli (Large-seeded) chickpea genotypes to water stress during seed filling: effects on accumulation of seed reserves and yield. J. Sci. Food Agric. 86, 2076–2082. doi: 10.1002/Jsfa.2574

[B84] Osakabe Y. Mizuno S. Tanaka H. Maruyama K. Osakabe K. Todaka D.. (2010). Overproduction of the membrane-bound receptor-like protein kinase 1, RPK1, enhances abiotic stress tolerance in arabidopsis. J. Biol. Chem. 285, 9190–9201. doi: 10.1074/Jbc.M109.051938 20089852 PMC2838338

[B85] Pan L. Zhang X. Wang J. Ma X. Zhou M. Huang L.. (2016). Transcriptional profiles of drought-related genes in modulating metabolic processes and antioxidant defenses in *Lolium Multiflorum* . Front. Plant Sci. 7. doi: 10.3389/Fpls.2016.00519 PMC4842912 27200005

[B86] Patel R. K. Jain M. (2012). NGS QC toolkit: a toolkit for quality control of next generation sequencing data. PloS One 7, E30619. doi: 10.1371/Journal.Pone.0030619 22312429 PMC3270013

[B87] Pribat A. Sormani R. Rousseau-Gueutin M. Julkowska Magdalena M. Testerink C.. (2011). A novel class of PTEN protein in arabidopsis displays unusual phosphoinositide phosphatase activity and efficiently binds phosphatidic acid. Biochem. J. 441, 161–171. doi: 10.1042/Bj20110776 21864294

[B88] Pujni D. Chaudhary A. Rajam M. V. (2007). Increased tolerance to salinity and drought in transgenic indica rice by mannitol accumulation. J. Plant Biochem. Biotechnol. 16, 1–7. doi: 10.1007/BF03321921

[B89] Purushothaman R. Krishnamurthy L. Upadhyaya H. Vadez V. Varshney R. (2016). Shoot traits and their relevance in terminal drought tolerance of chickpea *(Cicer Arietinum L.* ). Field Crops Res. 197, 10–27. doi: 10.1016/J.Fcr.2016.07.016 27698531 PMC5035057

[B90] Purushothaman R. Krishnamurthy L. Upadhyaya H. Vadez V. Varshney R. (2017). Genotypic variation in soil water use and root distribution and their implications for drought tolerance in chickpea. Funct. Plant Biol. 44 (2), 235–252. doi: 10.1071/FP16154 32480560

[B91] Remmers I. M. D’Adamo S. Martens D. E. de Vos R. C. H. Mumm R. America A. H. P.. (2018). Orchestration of transcriptome, proteome and metabolome in the diatom phaeodactylum tricornutum during nitrogen limitation. Algal Res. 35, 33–49. doi: 10.1016/j.algal.2018.08.012

[B92] Sen S. Chakraborty J. Ghosh P. Basu D. Das S. (2017). Chickpea WRKY70 regulates the expression of a homeodomain-leucine zipper (HD-zip) I transcription factor Cahdz12, which confers abiotic stress tolerance in transgenic tobacco and chickpea. Plant Cell Physiol. 58, 1934–1952. doi: 10.1093/Pcp/Pcx126 29016956

[B93] Sharma S. Mustafiz A. Singla-Pareek S. L. Shankar P. Srivastava Sopory S. K. (2012). Characterization of stress and methylglyoxal inducible triose phosphate isomerase *(Osctpi* ) from rice. Plant Sig. Behavior. 7, 1337–1345. doi: 10.4161/Psb.21415 PMC3493422 22902706

[B94] Shu J. Ma X. Ma H. Huang Q. Zhang Y. Guan M.. (2022). Transcriptomic, proteomic, metabolomic, and functional genomic approaches of brassica napus l. during salt stress. PloS One 17 (3), e0262587. doi: 10.1371/journal.pone.0262587 35271582 PMC8912142

[B95] Simova-Stoilova L. Demirevska K. Petrova T. Tsenov N. Feller U. (2009). Antioxidative protection and proteolytic activity in tolerant and sensitive wheat *(Triticum Aestivum L.*) varieties subjected to long-term field drought. J. Plant Growth Reg. 58 (1), 107–117. doi: 10.1007/S10725-008-9356-6

[B96] Sinha R. Irulappan V. Mohan-Raju B. Suganthi A. Senthil-Kumar M.. (2019). Impact of drought stress on simultaneously occurring pathogen infection in field-grown chickpea. Sci. Rep. 9, 5577. doi: 10.1038/s41598-019-41463-z 30944350 PMC6447570

[B97] Srivastava V. Obudulu O. Bygdell J. Löfstedt T. Rydén P. Nilsson R.. (2013). Onpls integration of transcriptomic, proteomic and metabolomic data shows multi-level oxidative stress responses in the cambium of transgenic hipi- superoxide dismutase populus plants. BMC Genom. 14, 893. doi: 10.1186/1471-2164-14-893 PMC3878592 24341908

[B98] Srivastava D. Verma G. Chauhan A. Pande V. Chakrabarty D. (2019). Rice *(Oryza Sativa L.*) tau class glutathione s-transferase *(Osgstu30*) overexpression in arabidopsis thaliana modulates a regulatory network leading to heavy metal and drought stress tolerance. Metallomics 11, 375–389. doi: 10.1039/C8mt00204e 30516767

[B99] Sudre D. Gutierrez-Carbonell E. Lattanzio G. Rellán-Álvarez R. Gaymard F. Wohlgemuth G.. (2013). Iron-dependent modifications of the flower transcriptome, proteome, metabolome, and hormonal content in an arabidopsis ferritin mutant. J. Exp. Bot. 64, 2665–2688. doi: 10.1093/Jxb/Ert112 23682113 PMC3697946

[B100] Tamiru M. Undan J. R. Takagi H. Abe A. Yoshida K. Undan J. Q.. (2015). A cytochrome P450, *Osdss1* , is involved in growth and drought stress responses in rice *(Oryza Sativa L.* ). Plant Mol. Biol. 88, 85–99. doi: 10.1007/S11103-015-0310-5 25800365

[B101] Thabet S. G. Moursi Y. S. Karam M. A. Graner A. Alqudah A. M. (2018). Genetic basis of drought tolerance during seed germination in barley. PloS One 13, E0206682–E0206682. doi: 10.1371/Journal.Pone.0206682 30388157 PMC6214555

[B102] Trapnell C. Pachter L. Salzberg S. (2009). Tophat: discovering splice junctions with RNA-seq. Bioinformatics 25 (9), 1105–1111. doi: 10.1093/Bioinformatics/Btp120 19289445 PMC2672628

[B103] Trapnell C. Roberts A. Goff L. Pertea G. Kim D. Kelley D. R.. (2012). Differential gene and transcript expression analysis of RNA-seq experiments with tophat and cufflinks. Nat. Prot. 7, 562–578. doi: 10.1038/Nprot.2012.016 PMC3334321 22383036

[B104] Tuteja N. (2007). Abscisic acid and abiotic stress signaling. Plant Signal Behav. 2, 135–138. doi: 10.4161/Psb.2.3.4156 19516981 PMC2634038

[B105] Tyanova S. Temu T. Cox J. (2016). The maxquant computational platform for mass spectrometry-based shotgun proteomics. Nat. Prot. 11, 2301–2319. doi: 10.1038/Nprot.2016.136 27809316

[B106] Varshney R. Song C. Saxena R. Azam S. Yu S. Sharpe A.. (2013a). Draft genome sequence of chickpea *(Cicer Arietinum*) provides a resource for trait improvement. Nat. Biotechnol. 31, 240–246. doi: 10.1038/Nbt.2491 23354103

[B107] Varshney R. K. Gaur P. M. Chamarthi S. K. Krishnamurthy L. Tripathi S. Kashiwagi J.. (2013b). Fast-track introgression of “QTL-hotspot” for root traits and other drought tolerance traits in JG 11, an elite and leading variety of chickpea. Plant Gen. 6, 1–9. doi: 10.3835/Plantgenome2013.07.0022

[B108] Varshney R. K. Thudi M. Nayak S. N. Gaur P. M. Kashiwagi J. Krishnamurthy L.. (2014). Genetic dissection of drought tolerance in chickpea *(Cicer Arietinum *L.). Theo. Appl. Genet. 127, 445–462. doi: 10.1007/S00122-013-2230-6 PMC3910274 24326458

[B109] Vessal S. Arefian M. Siddique K. H. M. (2020). Proteomic responses to progressive dehydration stress in leaves of chickpea seedlings. BMC Genom. 21, 523. doi: 10.1186/S12864-020-06930-2 PMC7392671 32727351

[B110] Walch-Liu P. Liu L. Remans T. Tester M. Forde B. (2006). Evidence that l-glutamate can act as an exogenous signal to modulate root growth and branching in arabidopsis thaliana. Plant Cell Physiol. 47, 1045–1057. doi: 10.1093/Pcp/Pcj075 16816406

[B111] Wang Z. Shi H. Yu S. Zhou W. Li J. Liu S.. (2019). Comprehensive transcriptomics, proteomics, and metabolomics analyses of the mechanisms regulating tiller production in low-tillering wheat. Theo. Appl. Genet. 132, 2181–2193. doi: 10.1007/s00122-019-03345-w 31020386

[B112] Williams M. E. Torabinejad J. Cohick E. Parker K. Drake E. J. Thompson J. E.. (2005). Mutations in the arabidopsis phosphoinositide phosphatase gene *SAC9* lead to overaccumulation of Ptdins(4,5)P2 and constitutive expression of the stress-response pathway. Plant Physiol. 138, 686–700. doi: 10.1104/Pp.105.061317 15923324 PMC1150389

[B113] Xu K. Chen S. Li T. Ma X. Liang X. Ding X.. (2015). *Osgras23* , a rice GRAS transcription factor gene, is involved in drought stress response through regulating expression of stress-responsive genes. BMC Plant Biol. 15 (141). doi: 10.1186/S12870-015-0532-3 PMC4465154 26067440

[B114] Yang S. Xu K. Chen S. Li T. Xia H. Chen L.. (2019). A stress-responsive bzip transcription factor *Osbzip62* improves drought and oxidative tolerance in rice. BMC Plant Biol. 19, 260. doi: 10.1186/S12870-019-1872-1 31208338 PMC6580479

[B115] Yang H. Zhang D. Li X. Li H. Zhang D. Lan H.. (2016). Overexpression of *Scaldh21* gene in cotton improves drought tolerance and growth in greenhouse and field conditions. Mol. Breed. 36, 34. doi: 10.1007/S11032-015-0422-2

[B116] Yao K. Wu Y. Y. (2016). Phosphofructokinase and glucose-6-Phosphate dehydrogenase in response to drought and bicarbonate stress At transcriptional and functional levels in mulberry. Russ. J. Plant Physiol. 63, 235–242. doi: 10.1134/S102144371602014X

[B117] Yoon Y. Seo D. Shin H. Kim H. J. Kim C. M. Jang G. (2020). The role of stress-responsive transcription factors in modulating abiotic stress tolerance in plants. Agronomy 10, 788. doi: 10.3390/Agronomy10060788

[B118] Yuan S. Kim S. Deng X. Hong Y. Wang X. (2019). Diacylglycerol kinase and associated lipid mediators modulate rice root architecture. New Phytol. 223, 261–276. doi: 10.1111/Nph.15801 30887532

[B119] Yun Z. Li T. Gao H. Zhu H. Gupta V. K. Jiang Y.. (2019). Integrated transcriptomic, proteomic, and metabolomics analysis reveals peel ripening of harvested banana under natural condition. Biomolecules. 9 (5), 167. doi: 10.3390/biom9050167 31052343 PMC6572190

[B120] Zeng J. Liu Y. Liu W. Liu X. Liu F. Huang P.. (2013). Integration of transcriptome, proteome and metabolism data reveals the alkaloids biosynthesis in *Macleaya Cordata* and *Macleaya Microcarpa* . PloS One 8, E53409. doi: 10.1371/Journal.Pone.0053409 23326424 PMC3541140

[B121] Zeng W. Peng Y. Zhao X. Wu B. Chen F. Ren B.. (2019). Comparative proteomics analysis of the seedling root response of drought-sensitive and drought-tolerant maize varieties to drought stress. Int. J. Mol. Sci. 20, 2793. doi: 10.3390/Ijms20112793 31181633 PMC6600177

[B122] Zhang M. Liu W. Bi Y. P. (2009). Dehydration-responsive element-binding (DREB) transcription factor in plants and its role during abiotic stresses. Yi Chuan = Hereditas 31, 236–244. doi: 10.3724/Sp.J.1005.2009.00236 19273435

[B123] Zhang R. X. Qin L. J. Zhao D. G. (2017). Overexpression of the osimp gene increases the accumulation of inositol and confers enhanced cold tolerance in tobacco through modulation of the antioxidant enzymes’ activities. Genes 8, 179. doi: 10.3390/Genes8070179 28726715 PMC5541312

[B124] Zhu Y. Liu Q. Xu W. Zhang J. Wang X. Nie G.. (2019). *De Novo* Assembly and discovery of genes that involved in drought tolerance in the common vetch. Int. J. Mol. Sci. 20, 328. doi: 10.3390/Ijms20020328 30650531 PMC6359484

