# Supplementary material for: Unravelling the molecular mechanism underlying drought stress response in chickpea via integrated multi-omics analysis
Source: Front Plant Sci. 2023 May 23;14:1156606. doi: 10.3389/fpls.2023.1156606 (PMC10242046; doi:10.3389/fpls.2023.1156606)
Supplement: Supplementary file 1 [file Presentation_1.pdf]

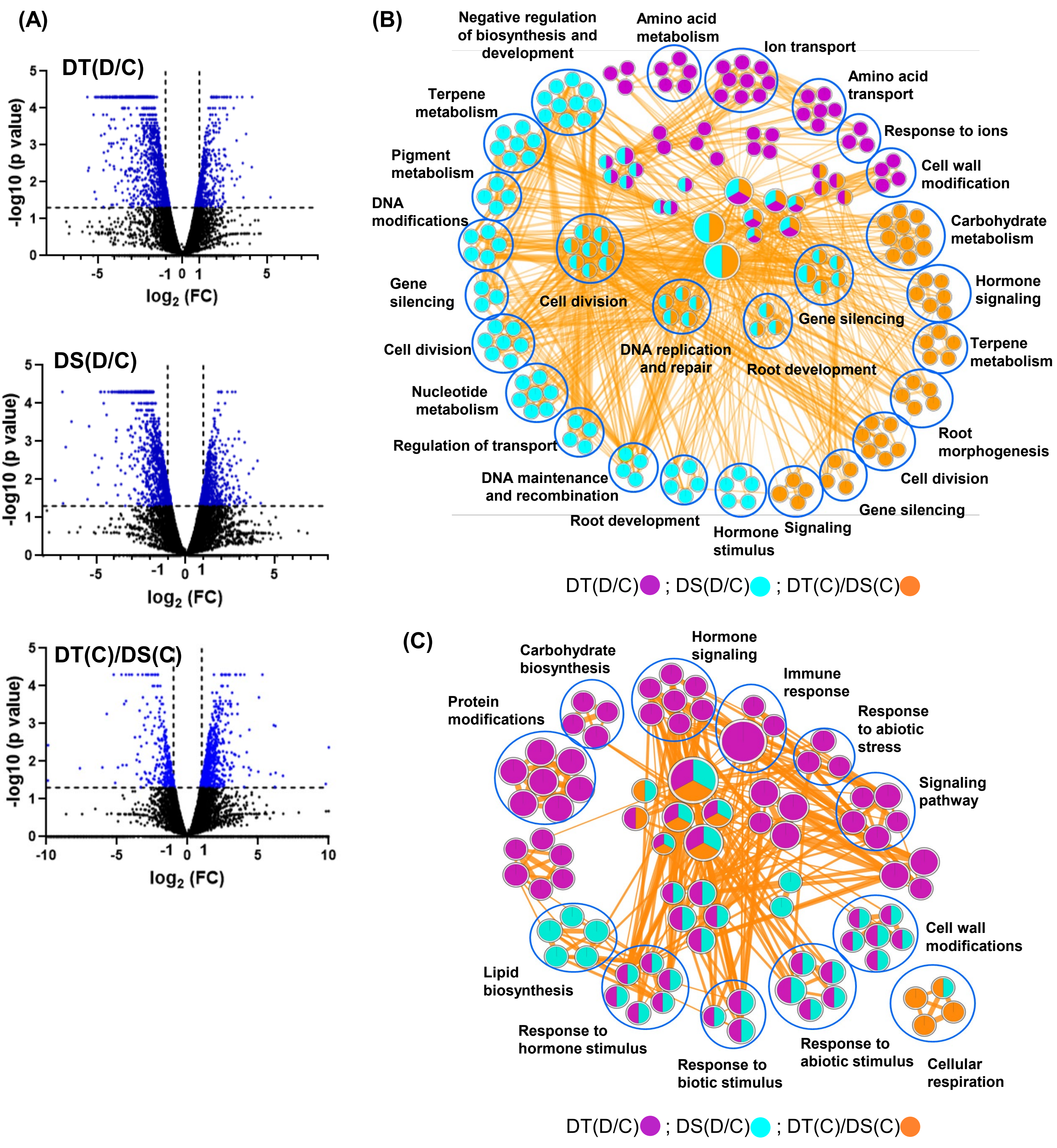

Figure S1. Analysis of differentially abundant transcripts (DATs) in drought-tolerant (DT) and drought-sensitive (DS) chickpea genotypes under control (C) and drought (D) conditions. DATs were determined for DT and DS genotypes under drought (D) as compared to control (C), DT(D/C) and DS(D/C), respectively, and under control condition for DT relative to DS genotype, [DT(C)/DS(C)]. **(A)** Volcano plots representing DATs for DT(D/C), DS(D/C) and DT(C)/DS(C) satisfying the criteria of $\log_2$ fold change (FC) of ≥ 1 (up-regulated) or ≤ -1 (down-regulated) at *P*-value ≤ 0.05. **(B, C)** Comparative GO enrichment analysis of up- **(B)** and down-regulated **(C)** genes for DT(D/C), DS(D/C) and DT(C)/DS(C). Significantly enriched GO terms (*P*-value ≤ 0.05) were used for the construction of enrichment maps. Node size represent the number of genes and node color represents *P*-value in the data set.

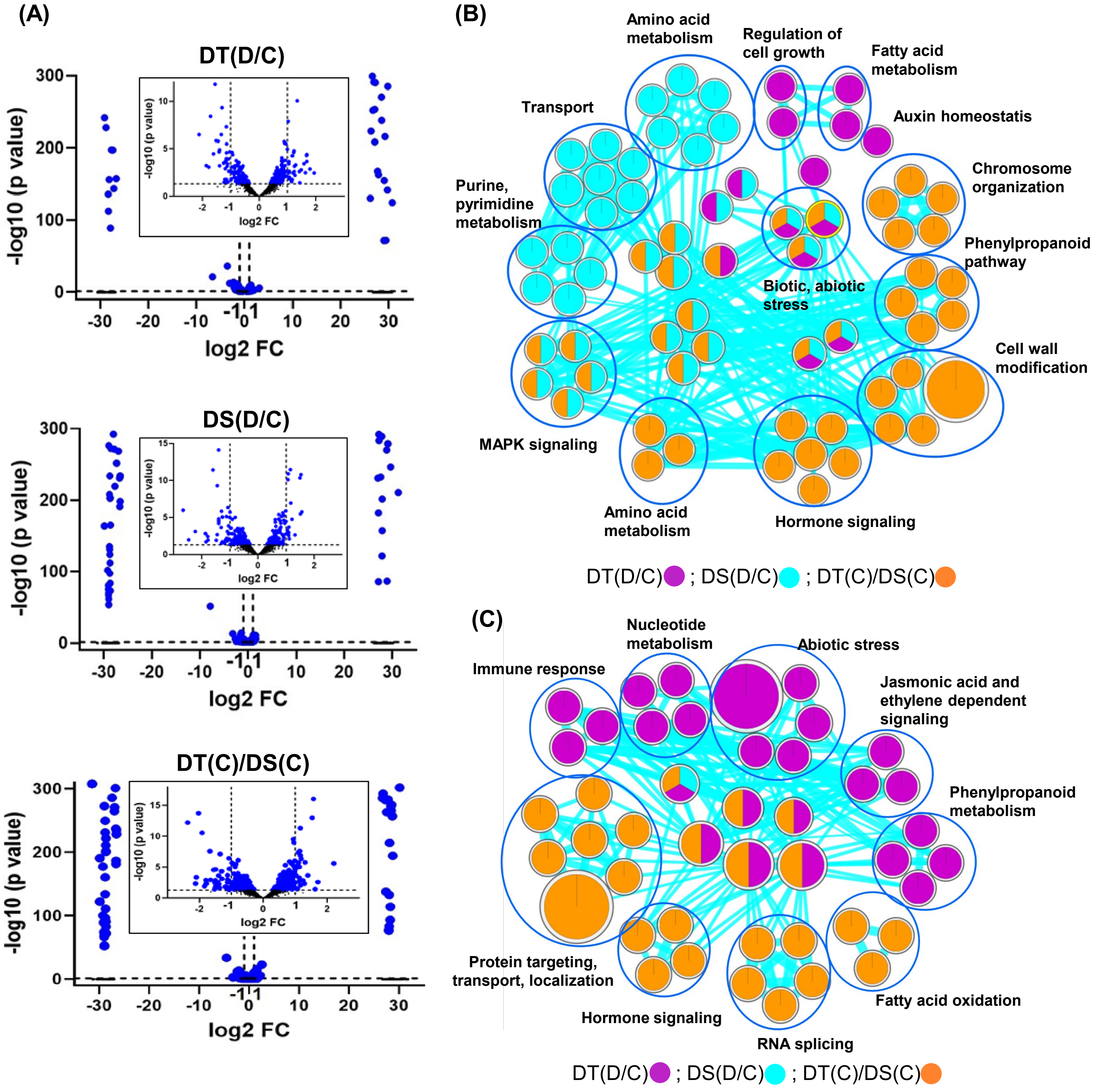

Figure S2. Analysis of differentially abundant proteins (DAPs) in drought-tolerant (DT) and drought-sensitive (DS) chickpea genotypes under control (C) and drought (D) conditions. DAPs were determined for DT and DS genotypes under drought (D) relative to control (C), DT(D/C) and DS(D/C), respectively, and under control condition for DT relative to DS genotype, [DT(C)/DS(C)]. **(A)**Volcano plot representing DAPs for DT(D/C), DS(D/C) and DT(C)/DS(C) satisfying the criteria of log_2 fold change (FC) value of ≥1 (up-regulated) or ≤ -1 (down-regulated) at *P*-value ≤ 0.05. Insets show the enlarged view of DAPs with FC value between -3 to 3. **(B, C)** Comparative GO enrichment analysis for up- **(B)** and **(C)** down-regulated proteins for DT(D/C), DS(D/C) and DT(C)/DS(C). Significantly enriched GO terms (*P*-value ≤ 0.05) were used for the construction of enrichment maps. Node size represent the number of proteins and node color represents *P*-value in the data set.

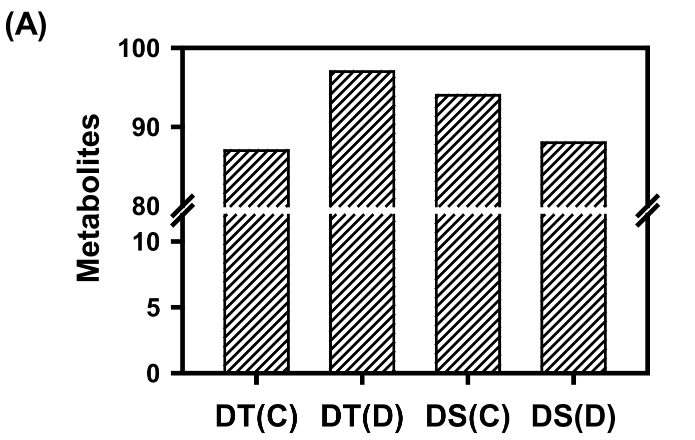

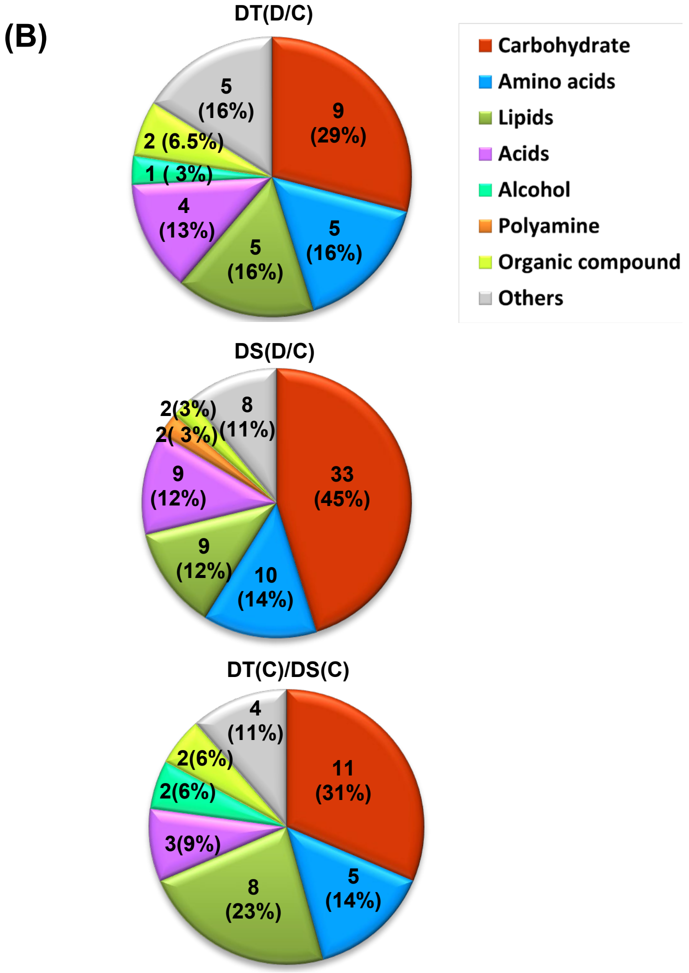

(C)

Class	Metabolite	DT(D/C)	DS(D/C)	DT(C)/DS(C)
Carbohydrates	D-Glucitol	3.67	3.05	-3.40
	L-Erythrulose	1.34	1.52	-
	Arabinonic acid	3.10	-3.61	-3.33
	D-Mannitol	2.56	-3.22	-2.94
	D-Psicofuranose	3.22	-	-
	D-Pinitol	1.37	-	-
	D-Trehalose	-	2.93	-3.84
	D-Turanose	-	-1.60	-2.02
	Inositol	-1.30	-2.09	-
	D-Galactose	-3.46	1.31	-
	D-Glucopyranoside	-3.19	-	3.72
	D-Lyxose	-	-4.40	-4.13
	Ketoglucose	-	-6.01	-5.68
	D-Glucopyranose	-	-	4.59
	D-Xylose	-	5.38	3.79
	D-Glucose	-	2.41	1.07
	Fucose	-	7.15	-
	Galactoside	-	5.23	-
	D-Altrose	-	5.85	-
	D-Erythrose	-	4.68	-
	D-Gluconic acid	-	4.85	-
	Anhydrohexitol	-	4.34	-
	Glyceric acid	-	4.39	-
	Palatinose	-	4.04	-2.67
	D-Tagatofuranose	-	3.47	-
	Hexopyranose	-	1.22	-
	Maltose	-	1.10	-
	D-Arabinose	-	-1.44	-
	Mannobiose	-	-1.09	-
	D-Sorbitol	-	-2.33	-
	D-Talose	-	-2.66	-
	Levoglucosan	-	-2.07	-
	D-Cellobiose	-	-3.86	-
	D-Mannose	-	-4.01	-
	Hexopyranoside	-	-3.88	-
	Glyceryl-glycoside	-	-3.19	-
	Myo-Inositol	-	-3.27	-
Amino acids	Lanthionine	1.42	2.01	-1.22
	L-Aspartic acid	1.59	1.62	-
	L-Threonine	-	-	-
	L-Oxoproline	-1.69	-	-1.54
	L-Glutamic acid	-5.04	-	-
	L-Phenylalanine	-	-2.63	-5.66
	L-Glycine	-	1.48	-4.22
	L-Isoleucine	-	-	-1.70
	L-Proline	-	4.21	-
	L-Threose	-	4.18	-
	L-Valine	-	1.63	-
	L-Asparagine	-	-2.96	-
	L-Leucine	-	-3.09	-
Lipids	Linoleoylglycerol	-	-4.07	-
	Linolenic acid	5.57	-6.68	-6.32
	Monopalmitin	4.26	-	-
	Arachidic acid	-4.73	-	-
	Behenic acid	-3.32	-3.80	-
	Adipic acid	-2.98	-	3.50
	Monostearin	-	4.44	-3.33
	Octadecadienoic acid	-	-	-3.49
	Stearic acid	-	-	3.25
	Myristic acid	-	-3.20	-1.55
	Palmitic acid	-	1.62	-
	Linoleic acid	-	-2.23	-
	Octadecanol	-2.31	-2.55	-
	Suberyl glycine	-	-7.32	-6.94
	Hydroxymethyl valerate	-	-3.32	-3.04
Acids	Isopropylmalic acid	5.20	-	-
	Phorbinepropanoic acid	3.63	-	-
	Oxalic acid	-3.93	-3.58	-
	Succinic acid	-1.19	-	-
	Biphenylcarboxylic acid	-	-10.18	-9.81
	Dihydroxybutanoic acid	-	-	4.09
	Propanedioic acid	-	-2.47	1.48
	Pimelic acid	-	7.21	-
	Ribonic acid	-	3.52	-
	Pipecolic acid	-	2.92	-
	Iodoacetic acid	-	2.68	-
	Ketoglutaric acid	-	-1.47	-
	Malic acid	-	-3.95	-
Alcohols	Methanol	-2.23	-	2.71
	Propanol	-2.37	-	2.85
Polyamines	Cadaverine	-	1.75	-
	Putrescine	-	5.21	-
Organic compounds	Pyrogallol	-5.01	-1.67	-
	Amphetamine	-	-7.35	-6.95
	Hexopyranuronate	-1.35	-	5.05
Others	Dimethyl palmitamine	4.36	-	-
	Biochanin A	2.94	-	-
	Acetin	2.66	1.59	-3.57
	Butanal	2.29	-	2.57
	Urea	1.45	-	-
	Ethylene brassylate	-	3.94	-
	Tromethamine	-	3.35	1.66
	Mercaptoethanol	-	-3.16	-2.89
	Erythronolactone	-	-2.59	-
	Ethanolamine	-	-2.44	-
	Tocopherol	-	-2.78	-
	Glycerol	-	-4.06	-

Log_2 FC

-10 10

Figure S3. Metabolites detected in drought-tolerant (DT) and drought-sensitive (DS) chickpea genotypes under drought (D) and control (C) conditions. **(A)** Bar diagram showing total number of high-confidence metabolites detected in each sample DT(C), DT(D), DS(C) and DS(D) after processing the raw data. **(B, C)** Differentially abundant metabolites (DAMs) were determined for DT and DS genotypes under drought (D) relative to control (C), DT(D/C) and DS(D/C), respectively, and under control condition for DT relative to DS genotype, [DT(C)/DS(C)]. DAMs detected in DT(D/C), DS(D/C) and DT(C)/DS(C) comparisons are given. **(B)** Pie charts representing numbers and percentage of metabolites in different classes in DT(D/C), DS(D/C) and DT(C)/DS(C) comparisons. **(C)** Heatmap showing all the DAMs belonging to different metabolic classes with their $\log_2$ fold change (FC) values.

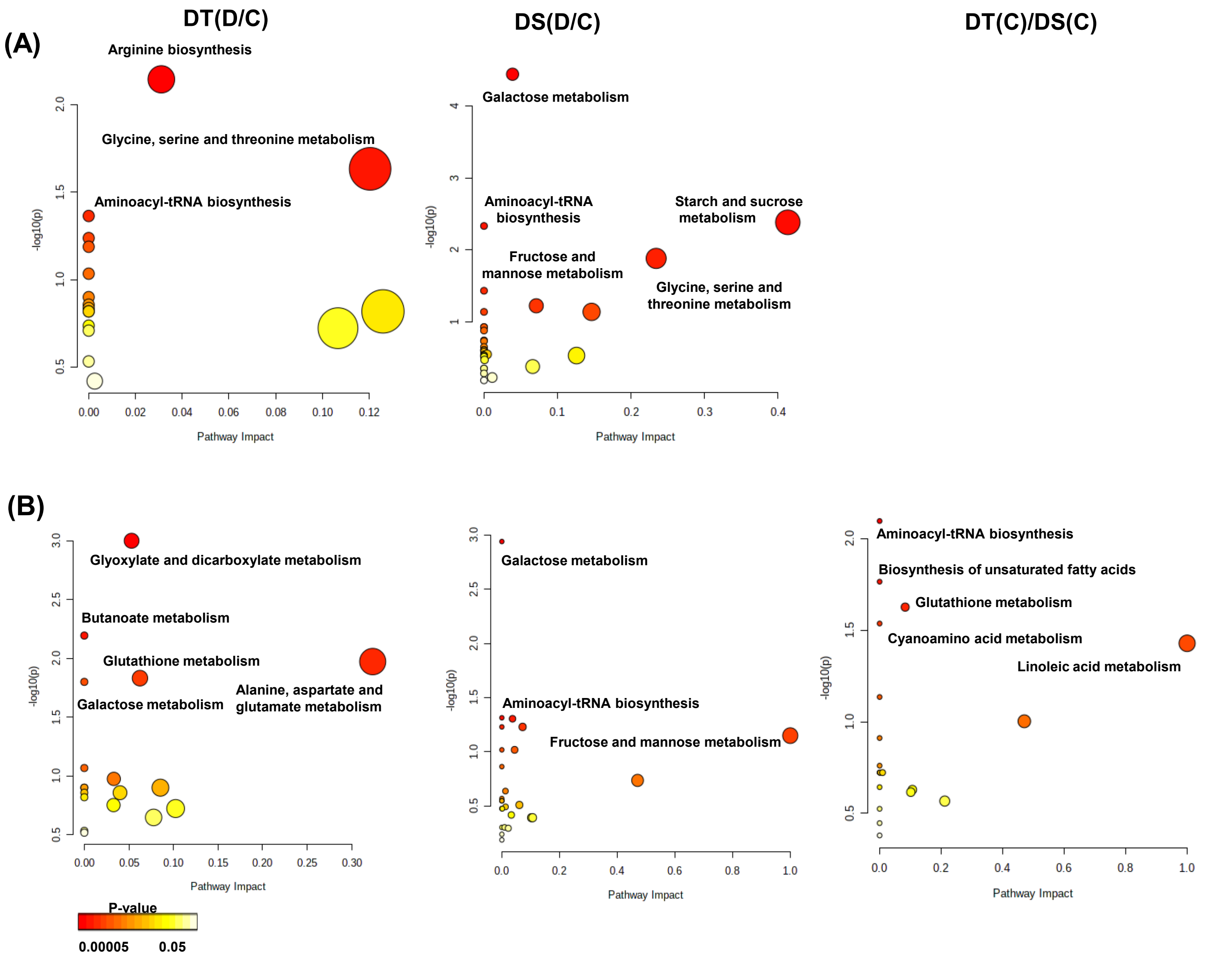

Figure S4. Pathway enrichment analysis of differentially abundant metabolites (DAMs) in drought-tolerant (DT) and drought-sensitive (DS) chickpea genotypes under drought (D) and control (C) conditions. DAMs were determined for DT and DS genotypes under drought (D) relative to control (C), DT(D/C) and DS(D/C), respectively, and under control condition for DT relative to DS genotype, [DT(C)/DS(C)]. Pathway enrichment analysis of up- **(A)** and down-regulated **(B)** metabolites for DT(D/C), DS(D/C) and DT(C)/DS(C) are shown. Bubble size represent pathway impact, whereas color represent –log10(p) value. High pathway impact and small *P*-value indicates higher significance.

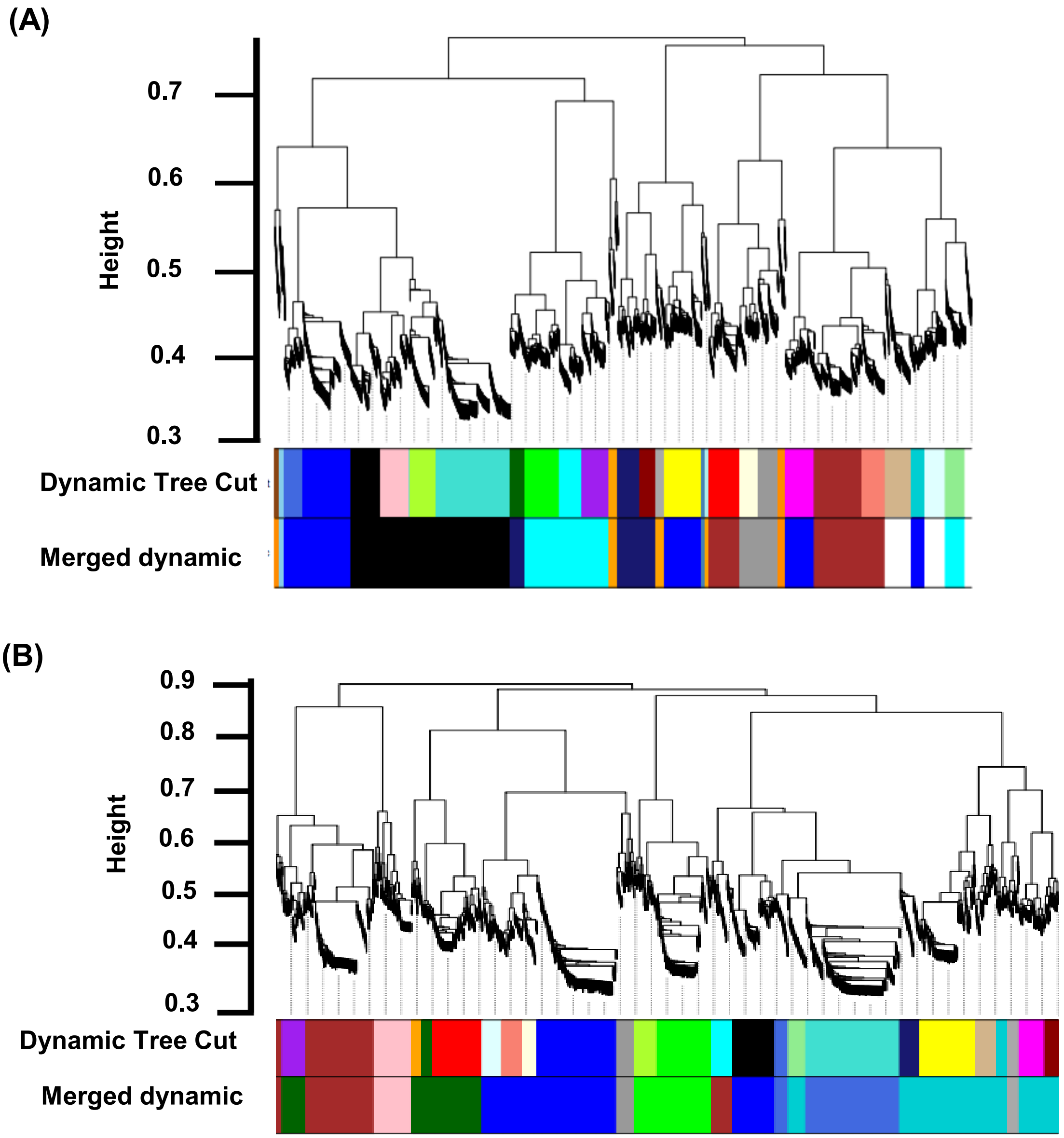

Figure S5. Cluster dendrograms showing co-expressed, **(A)** genes and gene modules of transcriptome, and **(B)** proteins and protein modules of proteome. Each branch denotes a gene/ protein, which are clustered according to dissimilarity measure (1-TOM) representing module. Original and merged modules are shown with their respective module colors. A total of 30 original modules were merged to obtain highly correlated, 11 and 9 modules for transcriptome and proteome, respectively.

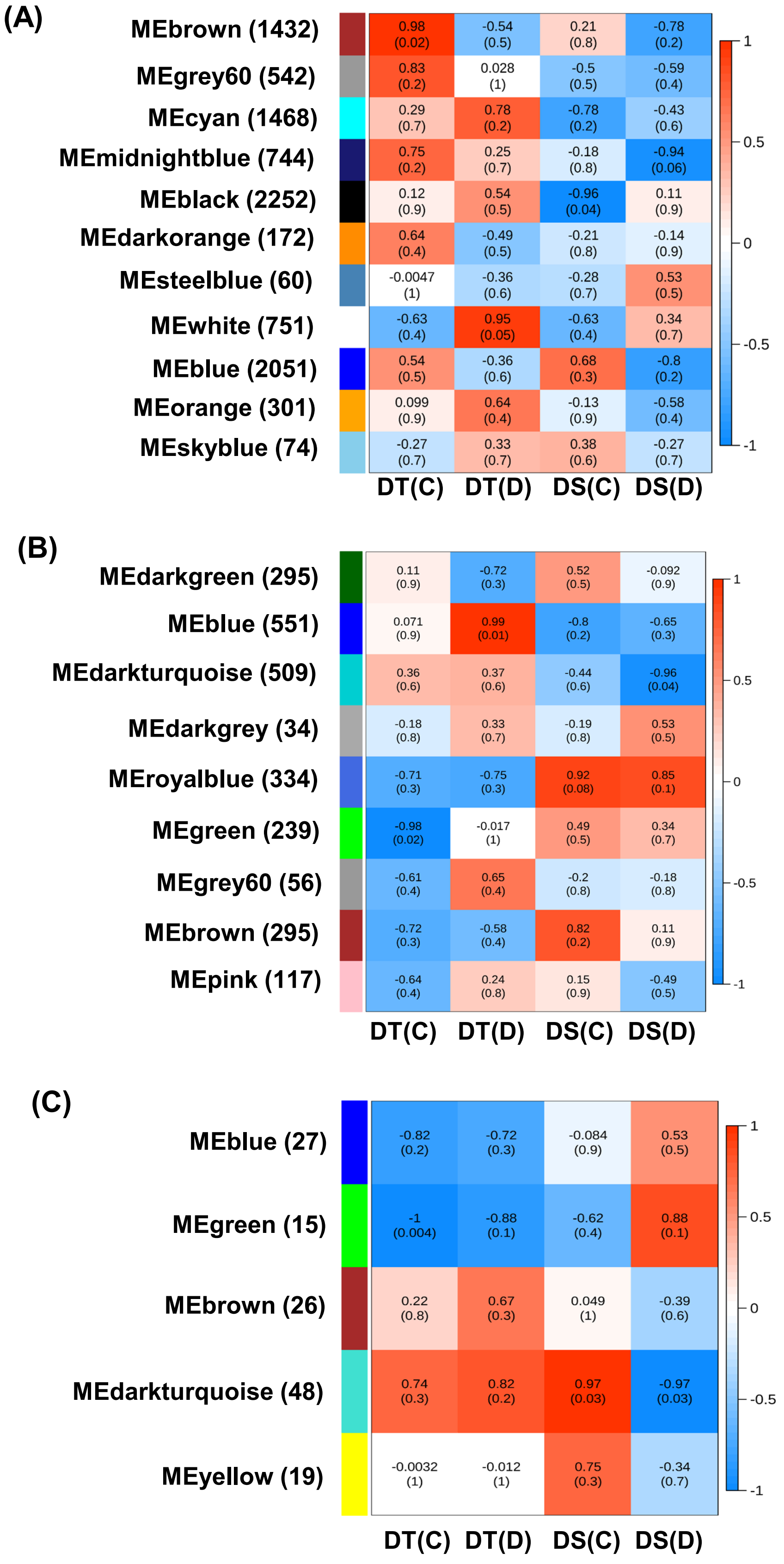

Figure S6. Co-expression analysis using weighted gene co-expression network analysis (WGCNA) for drought-tolerant (DT) and drought-sensitive (DS) chickpea genotypes under control (C) and drought (D) conditions. Module-sample relationship (MSR) matrix showing correlation between sample with **(A)** genes; **(B)** proteins and **(C)** metabolites. Correlation coefficients (r) are shown for each module-sample pair and *P*-value is mentioned in parenthesis. Color shows module-sample correlation as per scale indicated on right side. Total number of genes, proteins and metabolites in each module are shown in parenthesis.

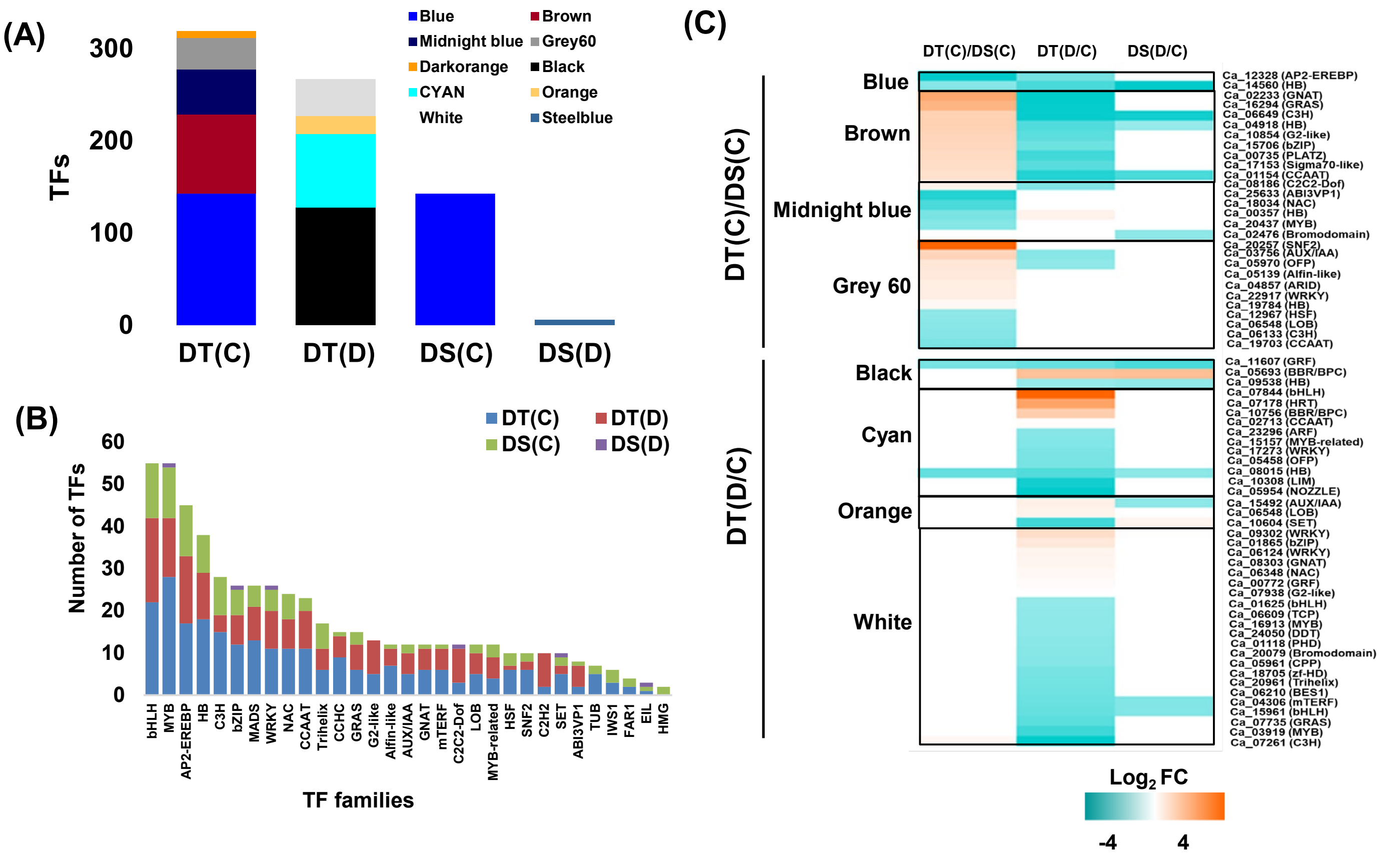

Figure S7. Transcription factors (TFs) encoding genes in drought-tolerant (DT) and drought-sensitive (DS) chickpea genotypes under control (C) and drought (D) conditions. **(A)** Bar diagram represents TFs identified in co-expressed modules of DT(C), DT(D), DS(C) and DS(D). (**B**) Top 20 TF families of DT(C), DT(D), DS(C) and DS(D) are shown. (**C**) Heatmap representing differentially abundant (DA) TFs encoding genes identified in DT(C)/DS(C) (blue, brown, midnight blue and grey60 modules) and DT(D/C) (black, cyan, orange and white modules). DA-TFs were identified with the $\log_2$ fold change (FC) value of ≥1 (up-) or ≤ -1 (down-regulated) at *P*- value ≤ 0.05.

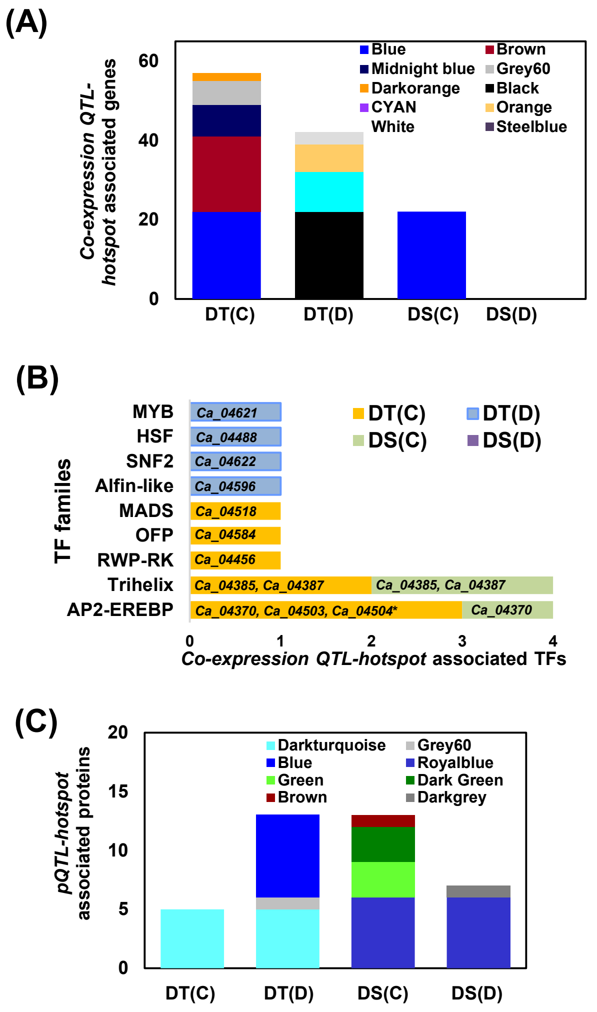

Figure S8. Quantitative trait loci-hotspot (*QTL-hotspot*) associated drought responsive genes, proteins and TFs in drought-tolerant (DT) and drought-sensitive (DS) chickpea genotypes under control (C) and drought (D). **(A)** Bar plots showing co-expression *QTL-hotspot* region (11, 284,553 bp to 14,082,277 bp) associated co-expressed genes in different modules of DT(C), DT(D), DS(C) and DS(D). **(B)** co-expression *QTL-hotspot* region associated TF families and related genes in DT(C), DT(D), DS(C) and DS(D). **(C)** Bar plots showing *pQTL-hotspot* region (11,396,081 to 14,093,770 bp) associated co-expressed proteins in different modules of DT(C), DT(D), DS(C) and DS(D).

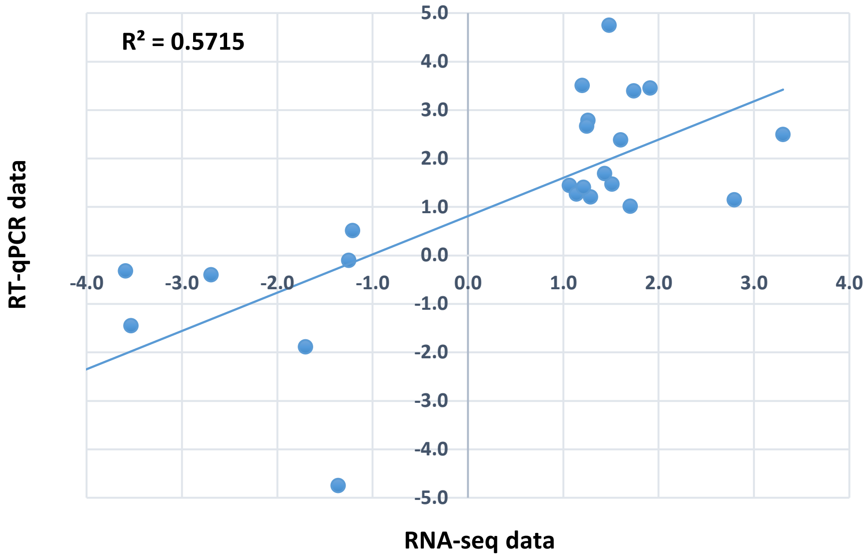

Figure S9. Figure showing the correlation between RT-qPCR and RNA-Seq data for selected candidate genes.
